# Supplementary material for: Whole-genome analysis of human embryonic stem cells enables rational line selection based on genetic variation
Source: Cell Stem Cell. 2022 Mar 3;29(3):472–486.e7. doi: 10.1016/j.stem.2022.01.011 (PMC8900618; doi:10.1016/j.stem.2022.01.011)
Supplement: Document S1. Figures S1–S5 [file mmc1.pdf]

Cell Stem Cell, Volume *29*

Supplemental Information

Whole-genome analysis of human embryonic stem cells enables rational line selection based on genetic variation

Florian T. Merkle, Sulagna Ghosh, Giulio Genovese, Robert E. Handsaker, Seva Kashin, Daniel Meyer, Konrad J. Karczewski, Colm O'Dushlaine, Carlos Pato, Michele Pato, Daniel G. MacArthur, Steven A. McCarroll, and Kevin Eggan

Supplementary figures and legends

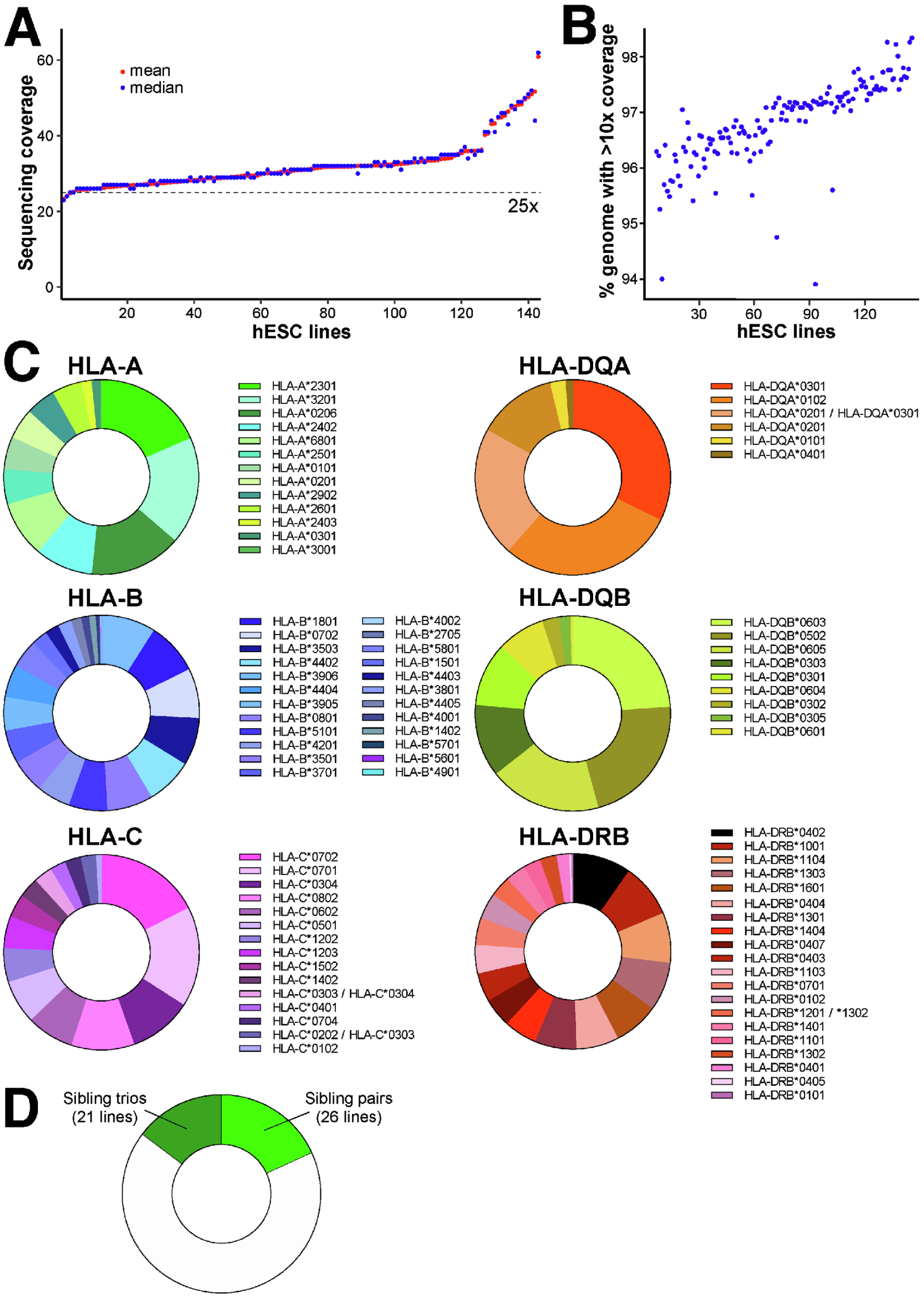

Figure S1: Sample sequencing coverage and diversity, related to main Figures 1 and 2. A) Whole genome sequencing coverage exceeded a mean and median of 25x for 140/143 human embryonic stem cell (hESC) lines, sorted by mean sequencing coverage. **B)** Percentage of the aligned genome sequenced to at least 10x mean coverage for 143 hESC lines sorted as in A. **C)** The human leukocyte antigen (HLA) haplotype diversity of 121 analyzed lines is consistent with their predominantly European origin. **D)** Approximately one third of sequenced hESC lines share sibling relationships.

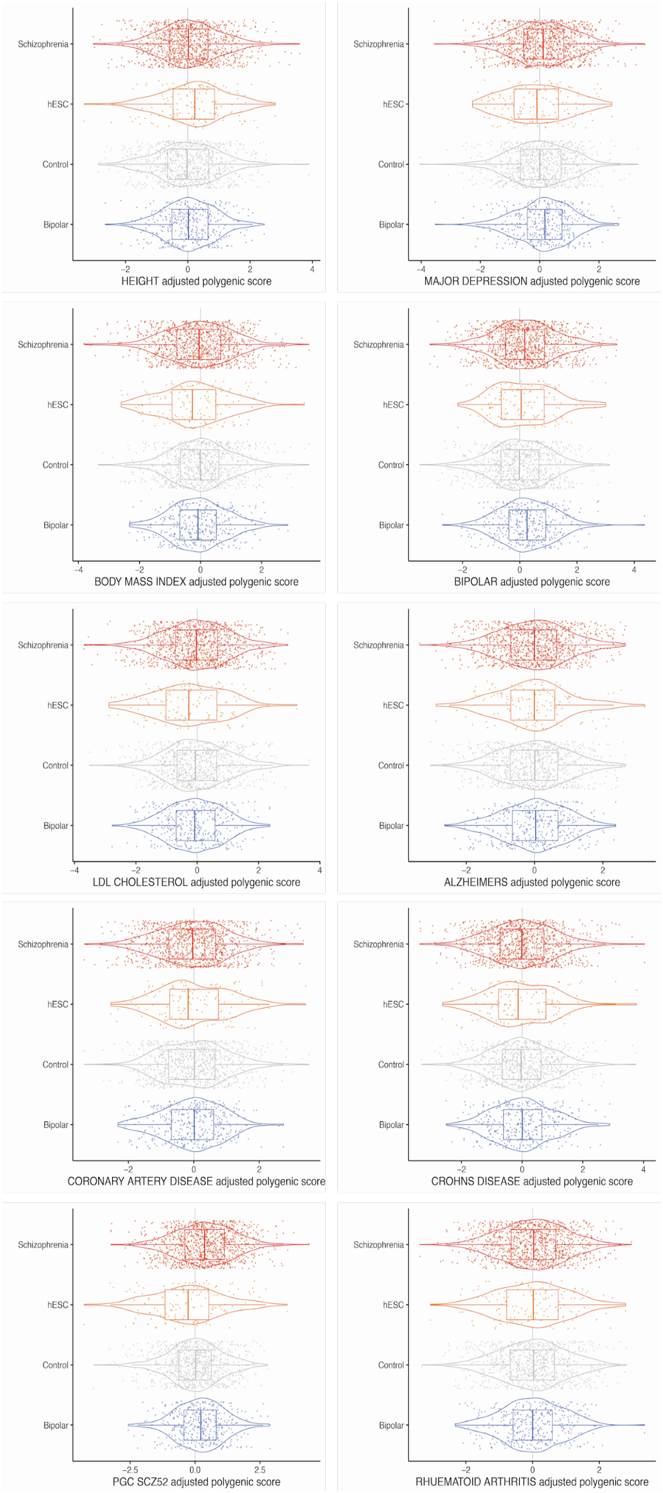

Figure S2: PRS distributions for hESCs and control samples, related to main Figure 3. Polygenic risk scores (PRS) were computed for hESCs and control samples from individuals with no diagnosed mental disorder or with either schizophrenia or bipolar disorder, normalized so that the control distribution had a mean of 0 and standard deviation (SD) of 1, and compared to reveal hESC lines that deviated more than 2 SD from the mean. Note that the only distribution that significantly deviates from the mean is the schizophrenia cohort when PRSs for schizophrenia were calculated (lower left). LDL, low density lipoprotein; PGC SCZ52, schizophrenia from the psychiatric genomics consortium.

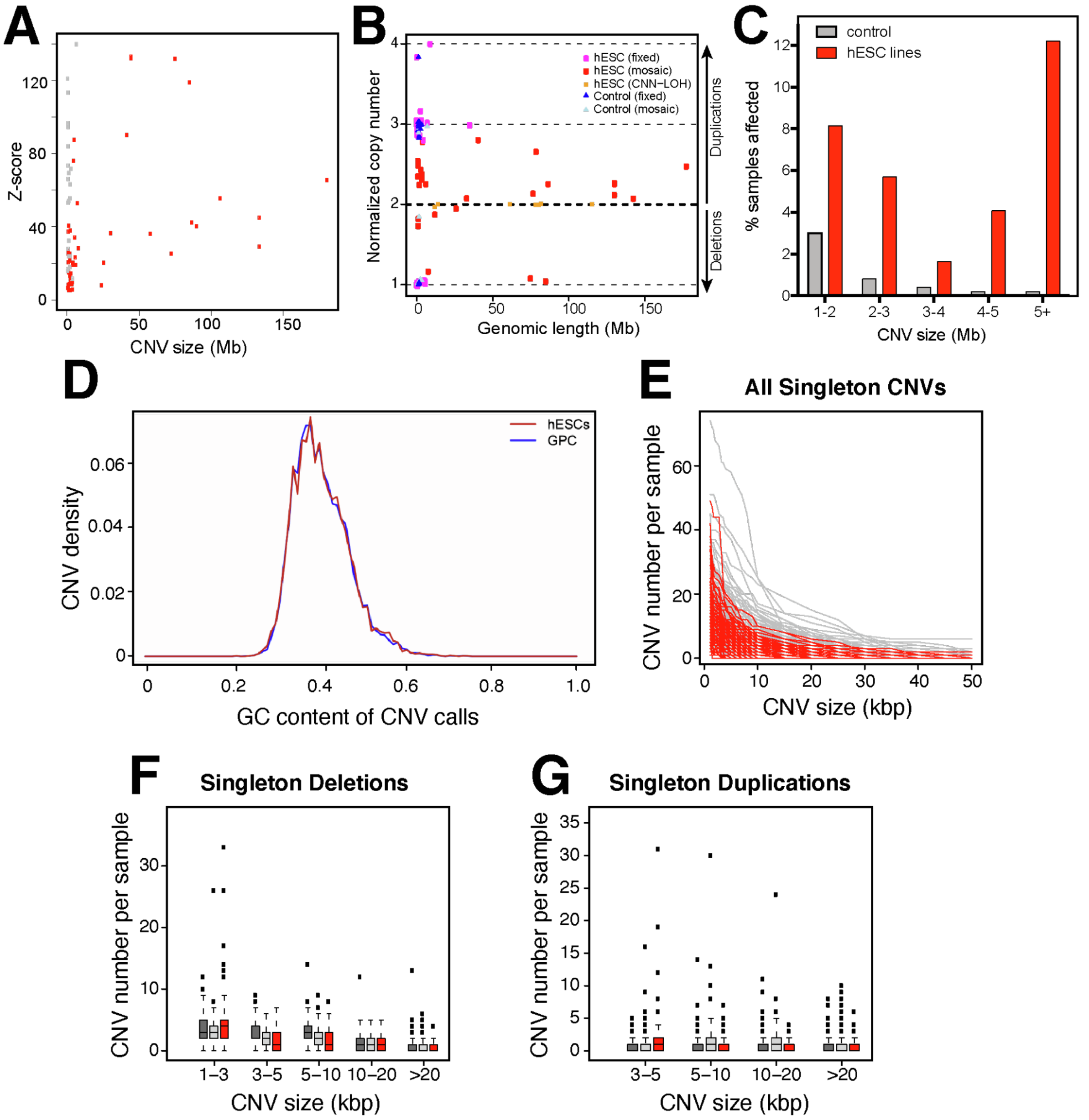

Figure S3: Structural genetic variant calling in hESCs, related to main Figure 4. A) Depth-of-coverage (DOC) analysis enables identification of structural variants over a wide range of sizes based on deviation from expected coverage from other samples considered in parallel (Z-score). Controls are shown in grey and hESCs are shown in red for variants >1 Mbp. **B)** CNVs from hESC or control samples that significantly ($P < 0.001$) deviated from a copy number of 1, 2, 3, or 4 were classified as mosaic. **C)** hESC lines show an excess of CNVs 1 Mbp or larger relative to similarly-sequenced control samples. **D)** Distribution of GC content in CNVs called in hESC WGS libraries prepared by tagmentation (red, hESCs) and CNVs called in control somatic cells from WGS libraries prepared by sonication (blue, GPC). **E)** Analysis of singleton CNVs suggests that hESC samples (red) do not carry an excess burden of potentially culture-acquired small CNVs relative to control populations (grey). **F,G)** Singleton deletions (F) or duplications (G) appearing only once in the dataset are not enriched in hESC lines (red) relative to control African American (dark grey) or Latino (light grey) samples.

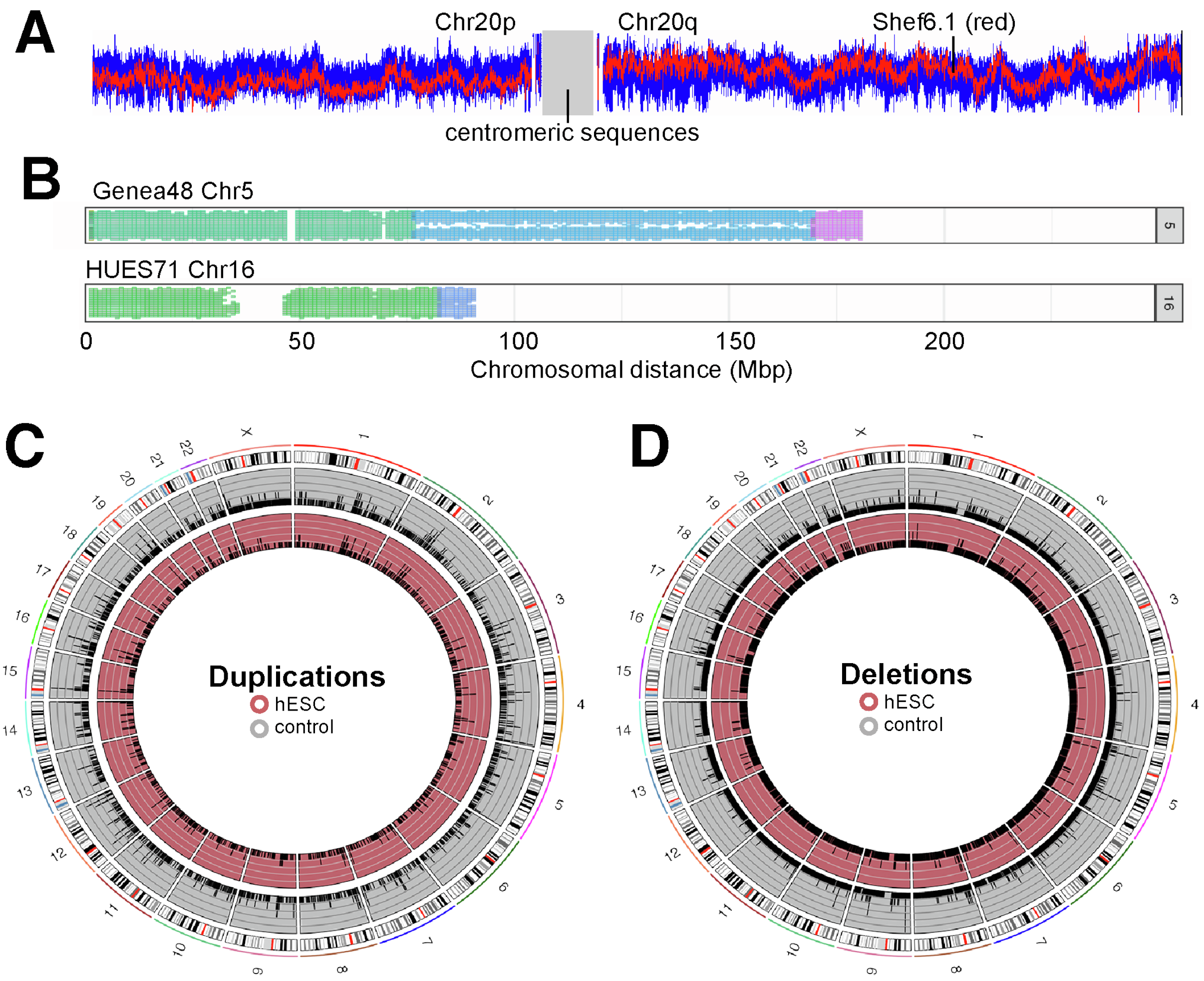

Figure S4: CNV calling and distribution in hESCs, related to main Figure 4. A) Normalized read DOC across chromosome 20 indicates loss of Chr20p and duplication of Chr20q in a subset of cells in hESC line Shef6.1 (red trace) relative to the other 120 cell lines (blue traces). The centromere is indicated in grey. **B)** Two cell lines show evidence for trisomy rescue since they contained genomic regions failed to phase when assuming a diploid model and gave patterns consistent with three distinct haplotypes rather than the expected two haplotypes over sections of chromosome 5 (1,853,207-76,720,244) for Genea48 (top) or chromosome 16 (start-82,687,797) for HUES71 (bottom). The boundaries of these regions likely correspond to recombination and nondisjunction events from meiosis I, which were then resolved in a subset of cells within the blastocyst or in cell culture leading to various levels of mosaicism of these haplotypes in the affected chromosomes (different colours). **C,D)** Small duplications (C) or deletions (D) in hESC lines (red) do not show clear evidence of recurrence relative to control samples (grey).

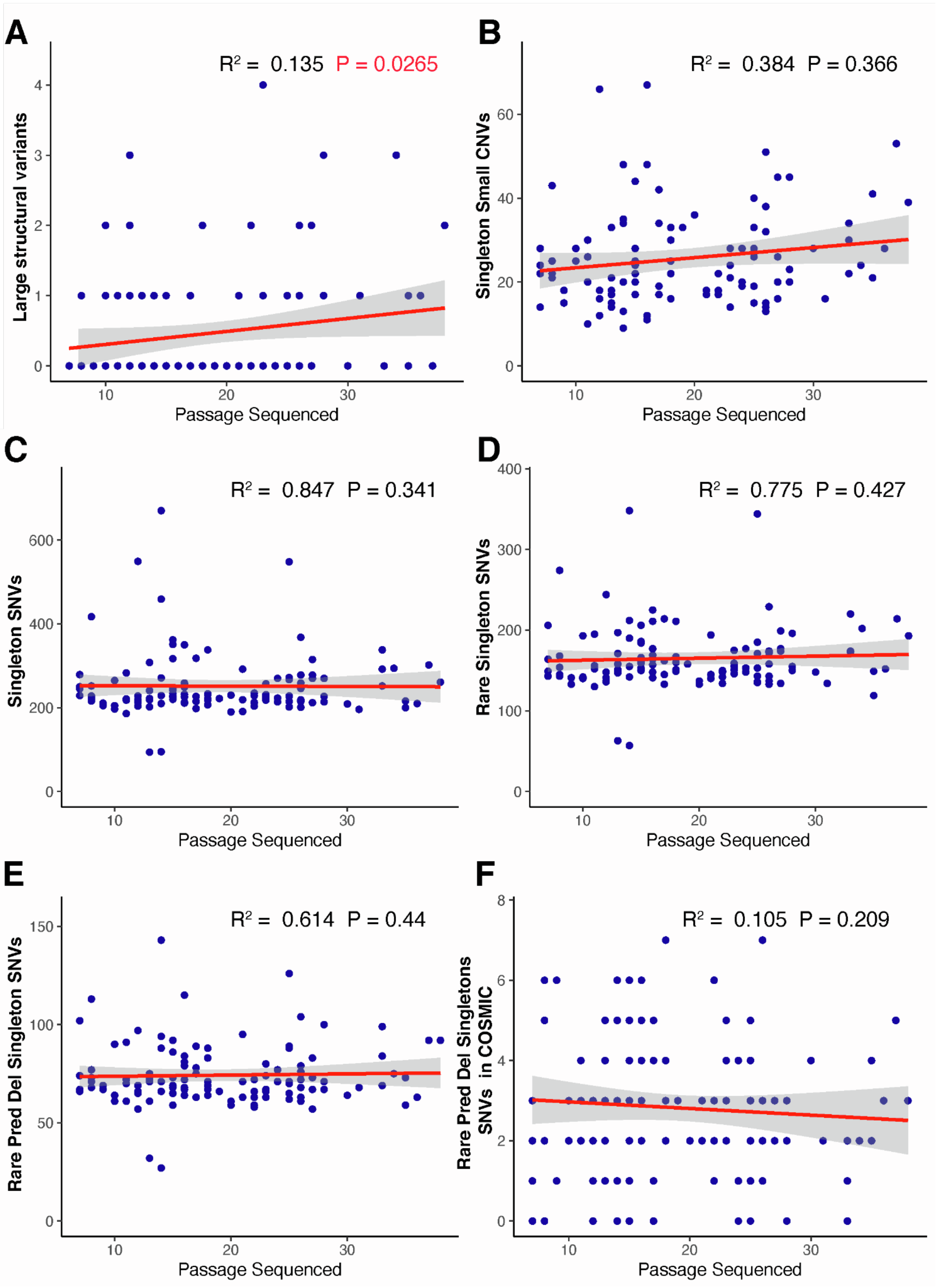

Figure S5: Linear regression across genetic variant types in hESCs, related to main Figures 4-6. The abundance of different classes of genetic variants identified in hESCs was compared to sex chromosome status, clinical-grade or research-grade, passage number at the time of sequencing, and principal components from SNP ancestry analysis. There is a positive correlation between passage number and the abundance of large structural variants per cell line **(A)**, but not for singleton small CNVs (**B**), singleton SNVs (**C**), rare singleton SNVs (**D**), likely deleterious rare singleton SNVs (**E**), or rare SNVs found in the COSMIC database (**F**).
