# Supplementary material for: Whole-genome analysis of human embryonic stem cells enables rational line selection based on genetic variation
Source: Cell Stem Cell. 2022 Mar 3;29(3):472–486.e7. doi: 10.1016/j.stem.2022.01.011 (PMC8900618; doi:10.1016/j.stem.2022.01.011)
Supplement: Document S2. Article plus supplemental information [file mmc6.pdf]

Cell Stem Cell

Resource

Whole-genome analysis of human embryonic stem cells enables rational line selection based on genetic variation

Graphical abstract

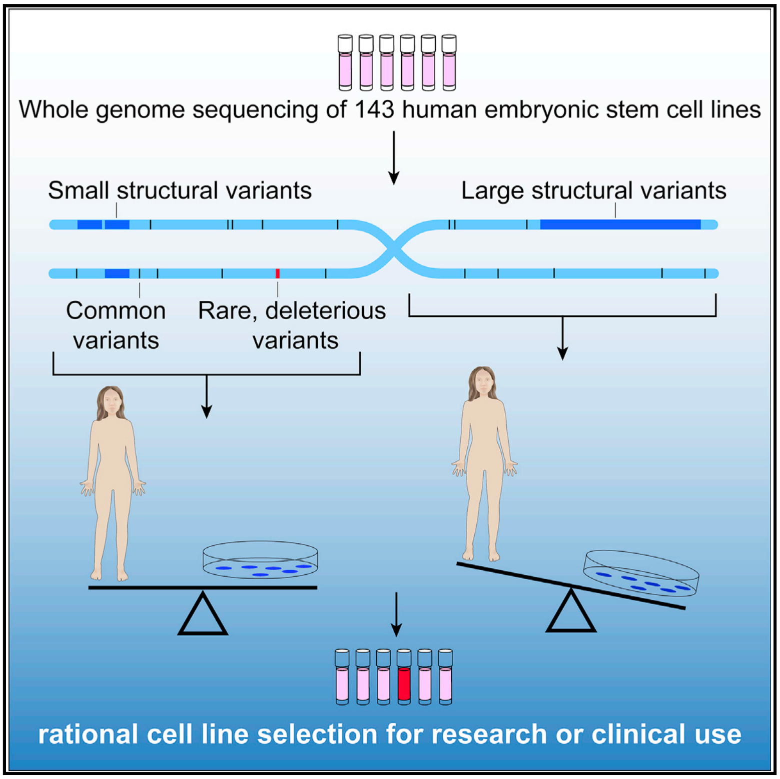

Highlights

- One-third of commonly used hESCs whose DNA we analyzed are one another's siblings
- hESCs have excess large structural variants but expected numbers of smaller variants
- Strong effect disease-associated mutations are present in specific hESC lines
- We provide user-friendly resources for sequence-guided rational stem cell line selection

Authors

Florian T. Merkle, Sulagna Ghosh, Giulio Genovese, ..., Daniel G. MacArthur, Steven A. McCarroll, Kevin Eggan

Correspondence

fm436@medschl.cam.ac.uk (F.T.M.), mccarroll@genetics.med.harvard.edu (S.A.M.), kevin.eggan@bmrn.com (K.E.)

In brief

Merkle and Ghosh et al. describe insights from the whole-genome sequences of commonly used human embryonic stem cell (hESC) lines. Analyses of these sequences show that while hESC genomes have more large structural variants than humans do from genetic inheritance, hESCs do not have an observable excess of finer-scale variants. However, many hESC lines contain rare loss-of-function variants and combinations of common variants that may profoundly shape their biological phenotypes. Thus, genome sequencing data can be valuable to groups selecting cell lines for a given biological or clinical application, and the sequences and analysis reported here should facilitate such choices.

Merkle et al., 2022, Cell Stem Cell *29*, 472–486
March 3, 2022 © 2022 The Authors. Published by Elsevier Inc.
https://doi.org/10.1016/j.stem.2022.01.011

CellPress

CellPress
OPEN ACCESS

Cell Stem Cell

Resource

Whole-genome analysis of human embryonic stem cells enables rational line selection based on genetic variation

Florian T. Merkle,[1,2,3,4,5,6,14,15,*] Sulagna Ghosh,[1,2,3,4,14] Giulio Genovese,[4,7,8] Robert E. Handsaker,[4,7,8] Seva Kashin,[4,7,8] Daniel Meyer,[4,8] Konrad J. Karczewski,[8,9] Colm O'Dushlaine,[4] Carlos Pato,[10,11] Michele Pato,[10,11] Daniel G. MacArthur,[7,8,12,13] Steven A. McCarroll,[4,7,8,*] and Kevin Eggan[1,2,3,4,*]
[1]Department of Stem Cell and Regenerative Biology, Harvard University, Cambridge, MA 02138, USA
[2]Department of Molecular and Cellular Biology, Harvard University, Cambridge, MA 02138, USA
[3]Harvard Stem Cell Institute, Cambridge, MA 02138, USA
[4]Stanley Center for Psychiatric Research, Broad Institute of MIT and Harvard, Cambridge, MA 02142, USA
[5]Wellcome – MRC Institute of Metabolic Science, University of Cambridge, Cambridge CB2 0QQ, UK
[6]Wellcome – MRC Cambridge Stem Cell Institute, University of Cambridge, Cambridge CB2 0AW, UK
[7]Department of Genetics, Harvard Medical School, Boston, MA 02115, USA
[8]Program in Medical and Population Genetics, Broad Institute of MIT and Harvard, Cambridge, MA 02142, USA
[9]Analytic and Translational Genetics Unit, Massachusetts General Hospital, Boston, MA 02114, USA
[10]Department of Psychiatry, Robert Wood Johnson Medical School, Rutgers University, New Brunswick, NJ 08901, USA
[11]Department of Psychiatry, New Jersey Medical School, Rutgers University, Newark, NJ 07103, USA
[12]Centre for Population Genomics, Garvan Institute of Medical Research, and UNSW Sydney, Sydney, NSW, Australia
[13]Centre for Population Genomics, Murdoch Children's Research Institute, Melbourne, VIC, Australia
[14]These authors contributed equally
[15]Lead contact
*Correspondence: fm436@medschl.cam.ac.uk (F.T.M.), mccarroll@genetics.med.harvard.edu (S.A.M.), kevin.eggan@bmrn.com (K.E.)
https://doi.org/10.1016/j.stem.2022.01.011

SUMMARY

Despite their widespread use in research, there has not yet been a systematic genomic analysis of human embryonic stem cell (hESC) lines at a single-nucleotide resolution. We therefore performed whole-genome sequencing (WGS) of 143 hESC lines and annotated their single-nucleotide and structural genetic variants. We found that while a substantial fraction of hESC lines contained large deleterious structural variants, finer-scale structural and single-nucleotide variants (SNVs) that are ascertainable only through WGS analyses were present in hESC genomes and human blood-derived genomes at similar frequencies. Moreover, WGS allowed us to identify SNVs associated with cancer and other diseases that could alter cellular phenotypes and compromise the safety of hESC-derived cellular products transplanted into humans. As a resource to enable reproducible hESC research and safer translation, we provide a user-friendly WGS data portal and a data-driven scheme for cell line maintenance and selection.

INTRODUCTION

Human pluripotent stem cells (hPSCs) can self-renew indefinitely while retaining the ability to differentiate into many cell types. These properties make hPSCs a powerful resource for studying early human development, disease modeling, and drug discovery and increasingly for developing candidate cell therapies (Avior et al., 2016; Merkle and Eggan, 2013; Trounson and DeWitt, 2016) (https://clinicaltrials.gov). However, the utility of human embryonic stem cells (hESCs) and human induced pluripotent stem cells (hiPSCs) for these applications can be compromised by mutations that affect their differentiation potential, cellular phenotypes, or clinical safety. The nature of such mutations has been studied using Giemsa-band karyotyping, fluorescent *in situ* hybridization, comparative genome hybridization arrays, and high-density single-nucleotide polymorphism (SNP) DNA microarrays that have a spatial resolution of >100 kbp (Draper et al., 2004; Laurent et al., 2011; Lefort et al., 2008; Maitra et al., 2005; Närvä et al., 2010) These and subsequent studies have revealed recurrent, culture-acquired structural genetic variants, including a common duplication at Chr20q11.21 that has been attributed to the gain of the anti-apoptotic gene *BCL2L1* (Avery et al., 2013; Nguyen et al., 2014). However, the cause and functional consequences of most mutations observed in hPSCs remain poorly understood, and as much as 99% of the genome of most hPSCs remains unexplored. Consequently,

This is an open access article under the CC BY license (http://creativecommons.org/licenses/by/4.0/).

CellPress
OPEN ACCESS

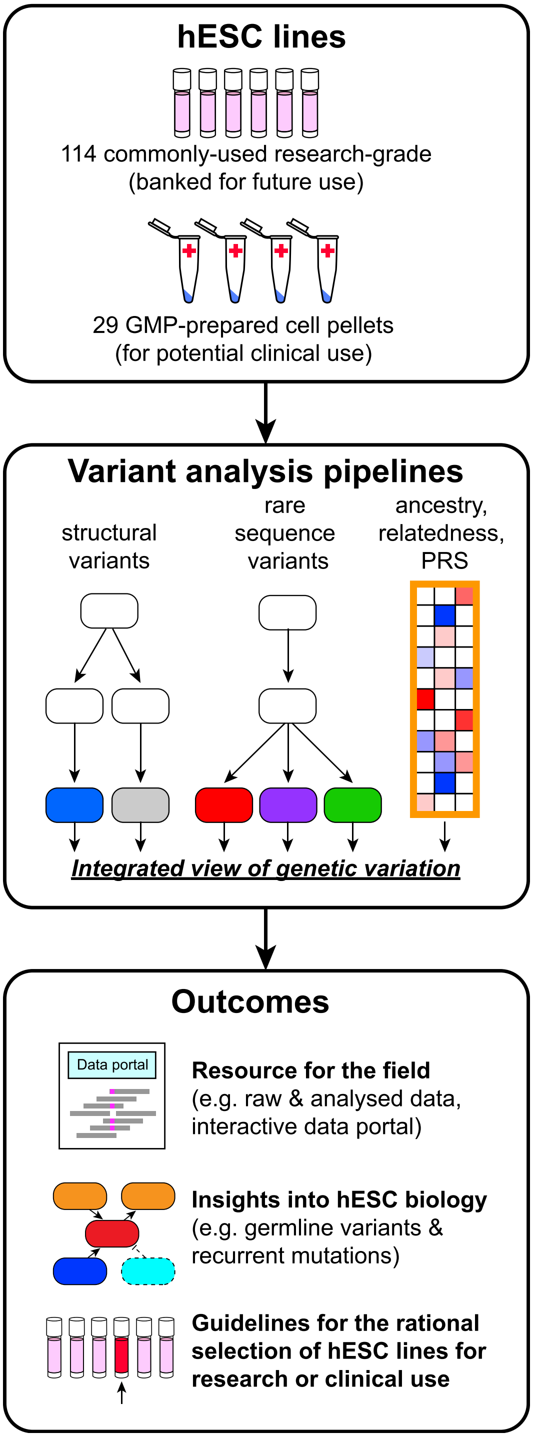

Figure 1. Study design and outputs

The genomic DNA of 143 human embryonic stem cell lines (hESCs) was analyzed by high-density SNP microarray and at the single-nucleotide level by WGS to call structural variants to a resolution of ~1 kbp, rare sequence variants associated with disease, and common sequence variants to reveal cell line ancestry, relatedness, and polygenic risk score (PRS). An integrated analysis of these data, which are provided as a resource to the field via an interactive data portal, yields insight into hESC biology and facilitates the rational selection of cell lines based on their genetic architecture. See also Table S1 and Figure S1.

hPSC lines are often viewed as being interchangeable, and lines for a given application are typically selected due to convenience or historical precedence rather than their intrinsic genetic suitability (Kobold et al., 2015).

To address this issue, we performed whole-genome sequencing (WGS, >25× coverage) and complementary high-density SNP genotyping of 143 hESC lines. We report our findings here as a resource. Though we confirmed that hESCs have an excess burden of large copy number variations (CNVs), we found that their overall burden of both single-nucleotide variants (SNVs) and small (<1 Mbp) CNVs resembled that of human populations, confirming hESCs as invaluable tools for studying human biology. Our analyses also bring to light recurrent acquired genetic variants that point to selective pressures exerted during self-renewal. These included a recurrent amplicon on Chr1q32.1, a copy-neutral loss of heterozygosity (CN-LOH) event at Chr9q, and small deletions encompassing the gene *EP300*, whose gene product stabilizes p53. Additional studies of SNVs identified from WGS data revealed deleterious variants in genes associated with cancer, infertility, and a variety of autosomal dominant diseases that could impact the phenotypic behavior of individual stem cell lines that harbor them. In order to allow researchers to query our data, we developed a user-friendly online data portal to further the goals of experimental reproducibility and the safety of future cell therapies.

RESULTS

hESC line selection and WGS

To gain insight into stem cell biology and to generate a valuable resource for the research and medical communities, we sequenced the whole genomes of 143 hESC lines that had been voluntarily deposited into the registry of hESCs maintained by the US National Institutes of Health (NIH) (http://grants.nih.gov/stem_cells/registry/current.htm) or that had been prepared for therapeutic applications (Figure 1; Table S1A). Genomic DNA from these cell lines was sequenced to a mean read depth of 32.2 (standard deviation [SD] 6.4, range 23.3 for HUES68 to 60.9 for KCL038, Figure S1A), with an average of 97% of the genome being sequenced at a minimum of 10× coverage (Figure S1B, STAR Methods).

hESCs are predominantly European and often share sibling relationships

Genetic background can be an important modifier of cellular phenotypes (Rouhani et al., 2014; Sittig et al., 2016). We therefore first investigated the genetic ancestry of hESC lines by drawing upon their ancestry-informative SNPs and comparing them with the diverse human populations sequenced in the 1000 Genomes Project (1000 Genomes Project Consortium, 2012; Sudmant et al., 2015). In agreement with previous studies (Mosher et al., 2010), principal component analysis (PCA) revealed that 93% (133/143) of sequenced hESC lines clustered together with samples of European ancestry (Figures 2A and 2B; Tables S1B and S1C). This finding was also reflected in their human leukocyte antigen (HLA) haplotypes (Nunes et al., 2014) (Figure S1C; Table S2A), which may be useful for groups seeking to match stem-cell-derived transplant and recipient HLA haplotypes.

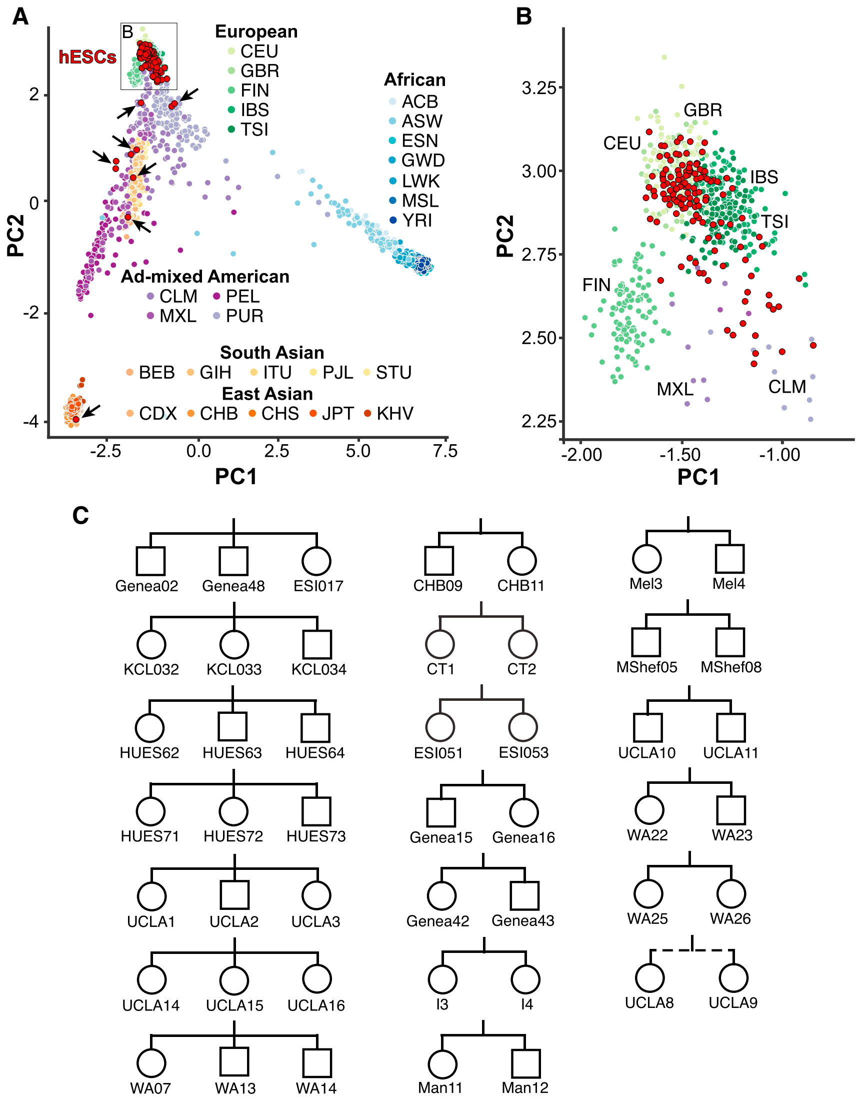

Figure 2. Ancestry and relatedness

(A) Principal component analysis illustrates the genetic ancestry of hESC lines (red) relative to individuals from diverse populations (three-letter codes) from the 1000 Genomes project. hESCs with ad-mixed European or non-European ancestry are indicated by arrows. PC, principal component.

(B) Magnification of the hESC cluster from (A) showing hESC line clustering with different European ancestries.

(C) Sibship pedigree of hESC lines, where squares denote male, circles denote female, and the dashed line denotes a half-sibling relationship. See also Table S2 and Figure S1.

Since multiple hESC lines can be derived from a cohort of embryos donated by a single couple undergoing assisted reproduction by *in vitro* fertilization (IVF) (Chen et al., 2009), we wondered how many sequenced hESC lines might exhibit genetic relatedness to one another. Upon analyzing shared SNP alleles (see STAR Methods), we found that 47/143 (33%) hESC lines shared a direct sibling relationship with another line we had sequenced, including seven sibling trios, 12 sibling pairs, and one half-sibling pair (Figures 2C and S1D; Tables S1A–S1C). Many of these sibling relationships were either unknown or unreported, and one sibling trio contained hESC lines from distinct providers (Genea02, Genea48, and ESI017) (Figure 2C). Upon contacting the providers, we learned that these cell lines were derived using materials from the same IVF clinic and that the sibling trio also included a fourth line (ESI014) not available for distribution. Awareness of these familial relationships should help guide experimental design, which in some contexts may aim to avoid shared genetic background, and in other contexts might exploit these properties to test genotype-phenotype relationships.

Common genetic variant contribution to risk of disease phenotypes

Common SNPs can impact the suitability of cell lines for modeling disease or transplantation. For example, a genetic variant in the gene *ABO* causes the O blood type (Yamamoto et al., 1990), a variant in *CCR5* renders cells resistant to HIV infection (Dean et al., 1996; Samson et al., 1996), and variants in the genes *APOE* (Corder et al., 1993) and *TREM2* (Guerreiro et al., 2013; Jonsson et al., 2013) are among the strongest known genetic contributors to cardiovascular disease (*APOE*) and Alzheimer's disease (AD, both *APOE* and *TREM2*). We therefore genotyped hESCs for these common variants and identified 22 cell lines with a "universal donor" O blood type, a cell line (Elf1) likely resistant to HIV infection, and three cell lines carrying the *APOE* "e4/e4" risk haplotype for cardiovascular disease and AD (Figures 3A–3C; Table S2B).

There is also accumulating evidence that the combined actions of thousands of common SNPs can contribute substantially to the risk of developing certain conditions, often conferring as much risk as large-effect (Mendelian) variants (Khera et al., 2018). The quantifiable contribution these SNPs confer can be represented through a polygenic risk score (PRS). To determine the currently calculable risk conferred by such variants to distinct disease phenotypes in each cell line, we computed PRSs for 18 distinct traits using data from well-powered genome-wide association studies (GWASs) adjusted for ancestry and normalized these scores to the distributions formed by larger numbers of similarly sequenced human samples (Bulik-Sullivan et al., 2015; Figures 3D and S2; Tables S2C and S2D). For each trait, we found one or more "outlier" hESC lines with a PRS at least two SDs from the mean. For example, WA21 has a high PRS for body mass index (BMI), suggesting that it might be predisposed to display obesity-relevant phenotypes if differentiated to relevant cell populations such as hypothalamic neurons (Merkle et al., 2015; Wang et al., 2015). Overall, 112/143 (78%) cell lines were outliers for at least one trait, and each cell line had a

CellPress
OPEN ACCESS

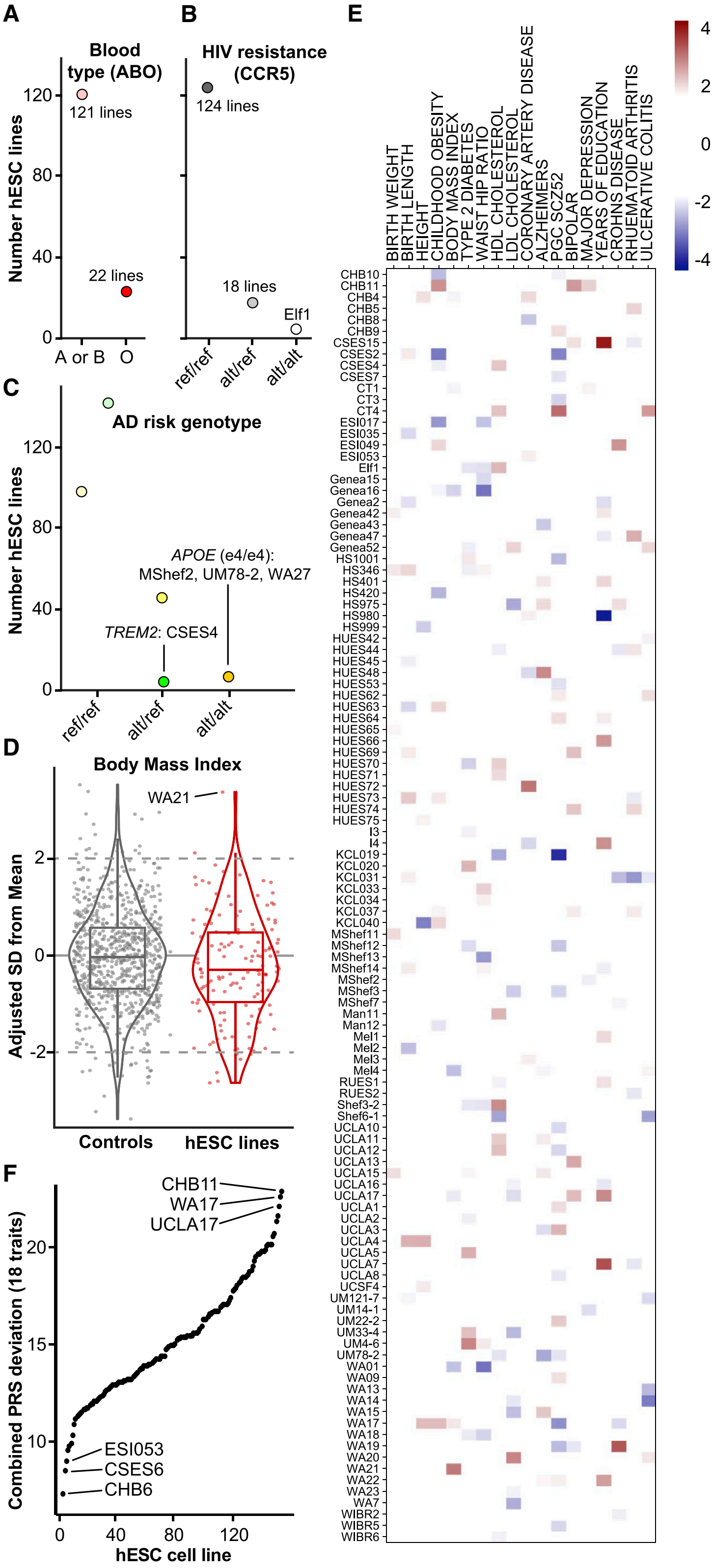

Figure 3. Disease risk from common genetic variants

(A–C) Genotyping results at SNPs indicative of (A) blood type (rs8176719 in *ABO*), (B) resistance to HIV infection (rs333 at CCR5), and (C) risk for Alzheimer's disease (AD, rs429358 and rs7412 in *APOE* in shades of orange, and rs75932628 in *TREM2* in shades of green). Ref, reference allele; alt, alternate (risk) allele.

(D) Distribution of polygenic risk scores (PRS) for control samples and hESCs reveals "outlier" samples with an SD of two or more.

(E) Heatmap of PRS for each of the 18 analyzed traits for cell lines with at least one "outlier" PRS.

(F) Combined PRS deviation from mean for each of the 18 analyzed traits. See also Table S2 and Figure S2.

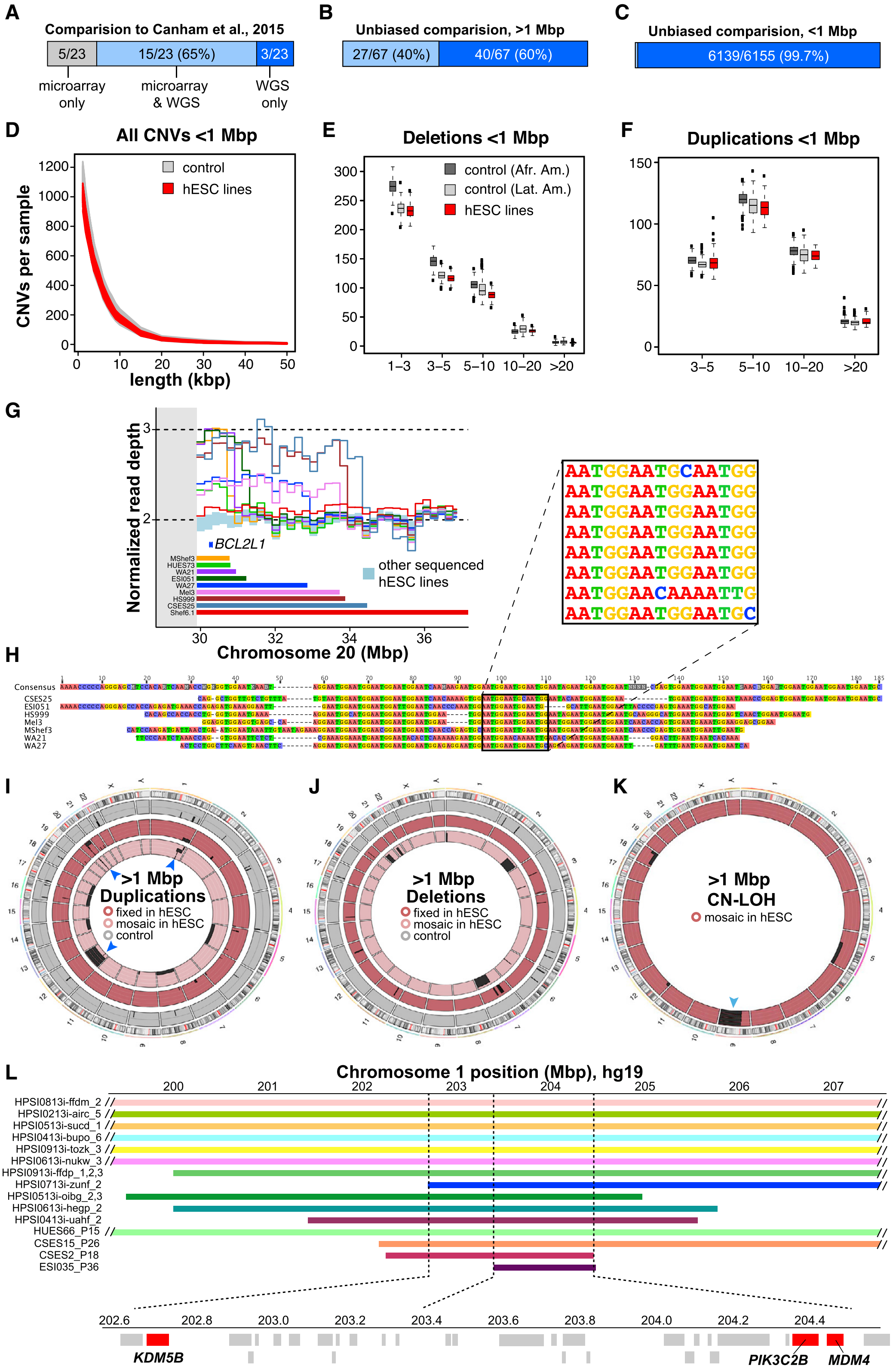

Figure 4. Structural variant calling from WGS data

(A) Comparison of structural variant calls in 22 lines shared between this study and a previous publication.

(B and C) Comparison of large (B) and small (C) structural variant calls made by microarray or WGS.

(legend continued on next page)

unique PRS fingerprint (Figure 3E). To identify hESCs that might make good "all-purpose control" cell lines, we ranked hESC lines by their combined absolute PRS across the 18 traits and identified cell lines with PRSs close to the population mean (Figure 3F; Table S2E).

Calling structural genetic variation from WGS data

Having established the genetic background of hESCs using common SNPs, we next analyzed their structural variants, which can affect the expression of tens to thousands of genes and significantly alter cellular phenotypes (Chiang et al., 2017). In particular, aneuploidy and large CNVs often contribute to disease (Henrichsen et al., 2009), and copy-neutral loss of heterozygosity (CN-LOH) events are frequently associated with cancer and can potently alter gene expression by affecting imprinted genes and unmasking disease-associated recessive mutations or risk alleles (Nicholls et al., 1989).

We reasoned that our WGS data with at least 25× mean sequencing depth of coverage should provide both superior spatial resolution and sensitivity for detecting small or mosaic CNVs in hESCs, compared with data from SNP DNA microarrays that sample only a small fraction of nucleotides. Indeed, we found that normalized read depth of coverage (DOC) analysis of WGS from 121 cell lines permitted the identification of deletions as small as ~1.1 kbp and duplications as small as ~2.8 kbp (Figure S3A). To complement this analysis, we identified heterozygous SNPs across the genome from WGS data and compared the sequencing depth of both alleles in all 143 hESC lines to call CNVs and CN-LOH events using the B allele frequency (BAF). We next split structural variants into "large" (>1 Mbp) and "small" (<1 Mbp) categories, revealing 66 distinct fixed and mosaic large structural variants affecting nearly a third of hESC lines (46/143, 32%; Figures S3B and S3C; Tables S3A and S3B).

To test the accuracy and sensitivity of our approach, we compared our WGS structural variant calls with a published SNP microarray-based study (Canham et al., 2015) that included 22 of the cell lines we subsequently sequenced. We found that WGS confirmed most of these variants, allowed CNV borders to be more accurately mapped, and revealed previously unascertained structural variants (Figure 4A; Tables S3C and S3D). To broaden this comparison, we analyzed identical genomic DNA samples from 121 hESC lines by both DOC and BAF analysis of WGS data and high-density SNP microarrays (Infinium PsychArray, > 500,000 probes). Of the large variants (>1 Mbp) observed by analyzing WGS data in these 121 lines, 41/58 (71%) were also called by PsychArray (Figure 4B; Table S3B). Together, these results suggest that analysis of WGS data has substantially improved the utility for calling large structural variants relative to microarrays and confirms and extends previous reports (International Stem Cell Initiative et al., 2011; Baker et al., 2016; Draper et al., 2004; Laurent et al., 2011; Lefort et al., 2008; Närvä et al., 2010) that hESCs carry an excess burden of large structural variants compared with somatic human cells (Figure S3C; Tables S3A and S3B).

Frequency of small CNVs in hESCs

Since large structural variants were relatively common in hESCs, we wondered whether the hESCs we sequenced might also carry an excess burden of small CNVs, which have not yet been comprehensively examined. To address this question, we filtered out genomic regions containing CNVs >1 Mbp from affected samples and studied the residual whole genomes of 121 hESCs alongside 234 comparably prepared control whole-genome sequences from human blood samples (Pato et al., 2013) for CNVs between 1.1 kbp and 1 Mbp. We did not classify small CNVs as fixed or mosaic due to the difficulty of distinguishing between integer and fractional changes in copy number at these length scales. We observed 6,155 unique CNVs (Table S3E), many of which were shared across cell lines, leading to an average of 999 ± 66 small CNVs per hESC sample (Table S3F). The number of small CNVs called from WGS data vastly exceeded the number of CNVs that could be called from PsychArray DNA microarray data from these same 121 hESC lines (16/6155, 0.3%, Figure 4C). To validate CNV calls, we identified those that contained two or more PsychArray probes (1024/6155, 17%) and compared probe intensities across all samples using an intensity ranked sum (IRS) test previously used to establish CNV false discovery rates (FDRs) (Handsaker et al., 2015; Mills et al., 2011). We observed an overall FDR of 5.7% (Table S3G), indicating that the vast majority of tested CNVs were independently validated.

Since hPSCs are highly proliferative and rates of DNA replication are uneven across the genome, WGS data can be used to gain insight into the biology of human DNA replication timing (Ding et al., 2021). If the small CNVs we identified corresponded to replication forks, we would expect them to be found in regions of high guanine-cytosine (GC) content—and to be found in excess in hESCs relative to more slowly proliferating samples. We therefore jointly called CNVs in hESC genomes and similarly sequenced primary human blood samples and found that CNVs called in both hESC and control samples had a similar CG content (Figure S3D), genomic distribution, and frequency at all length scales tested (Figures 4D–4F). Next, we asked whether any small CNVs might be culture acquired and restricted our analysis to CNVs present just once in the combined dataset of hESCs and somatic cell control samples. We found that both hESC and control samples harbored an average of 21 ± 11 of

(D) Length distribution of small CNVs in hESCs and controls.

(E and F) At length scales of ~1.1 kbp to 1 Mbp, the frequency of deletions (E) and duplications (F) in hESCs resembles that seen in control cells from African American (Afr. Amer.) and Latin American (Lat. Amer.) ancestries. Values on the x axis of 1–3 and 3–5 refer to 1.117–2.75 kbp and 2.75–5 kbp, respectively.

(G) Recurrent duplications of Chr20q11.21 encompassing the anti-apoptotic gene *BCL2L1* extend from the pericentromeric (gray) region, with each cell line (colored bars and lines) having distinct distal breakpoints.

(H) Alignment of sequencing reads flanking the distal Chr20q11.21 duplication breakpoint reveals a shared $(AATGG)_n$ motif.

(I–K) Circular ideograms of fixed (darker shades) and mosaic (lighter shades) duplications (I), deletions (J), and CN-LOH events (K) in 121 hESC lines (red shades) and 500 similarly sequenced controls (gray). Genomic regions with at least four recurrent events in hESCs are indicated by blue arrowheads. (L) A minimally duplicated region on Chr1q32.1 harbors candidate genes (red) among other coding genes (light gray). See also Table S3 and Figures S3 and S4.

such singleton CNVs (Figures S3E–S3G), suggesting that hESCs and human populations have a comparable burden of these structural variants.

Location and potential roles of structural variants in hESCs

Some structural variants recur in hESCs, and to gain insight into the underlying mechanisms we examined the well-studied Chr20q11.21 region in which duplications extend from a centromeric region out to the long arm of Chr20 (International Stem Cell Initiative et al., 2011). We observed duplications in 11/143 (8%) cell lines (Figure 4G), including one additional likely instance of isochromosome 20 (Figure S4A). This duplication probably confers a selective advantage since it contains the anti-apoptotic gene *BLC2L1* (Avery et al., 2013; Nguyen et al., 2014), although it remains unclear as to why this duplication recurs more often than other regions that harbor similar anti-apoptotic genes or proto-oncogenes. We mapped the Chr20q11.21 CNV breakpoints and found that distal breakpoints were unique for each cell line and that most of them shared a common centromere-like HSAT3 (GGAAT)n microsatellite repeat motif (Figure 4H). This motif is commonly seen on Chr20q11 (Altemose et al., 2014). As described elsewhere (Halliwell et al., 2021), this result suggests that Chr20q11.21 is prone to homology-based structural instability, which might explain its frequent recurrence in hPSCs.

To better understand the potential functional consequences of other large structural variants, we mapped them to the genome (Figures 4I–4K). We found that 15/143 (10%) hESC cultures contained aneuploid cells, all but one of which involved chromosomal gain, and were predicted to be present at a cellular fraction of 4%–66% (Table S3B). We also observed eight large, fixed duplications and 19 more that were mosaic or present in a fraction of cells (see STAR Methods), as well as six large fixed deletions and six large mosaic deletions (Table S3B). We also found that 12/143 (8%) hESC lines carried CN-LOH events in a subset of cells, of which five involved the entire q arm of chromosome 9 (Figure 4K). Since we had previously observed this structural variant arising *de novo* upon gene editing (Kiskinis et al., 2014), it is likely to be recurrently culture acquired (Figure 6B). To map the genetic elements that might be responsible for the recurrent duplication at Chr1q (Figures 4I and 4L), we identified 11 hiPSC lines with duplications over this interval in the HipSci resource (Kilpinen et al., 2017) (data accession: EGAD00010001147), that when combined with our hESC lines revealed a minimally duplicated sub-region spanning approximately chromosome 1 position 203,408,100 to 204,572,300 (hg19 assembly), corresponding to the cytogenetic location Chr1q32.1 (Figure 4L). This region contains candidate genes worthy of future investigation that may confer selective advantage when duplicated, including the p53 regulator *MDM4* (Francoz et al., 2006; Figure 6B; Table S3H).

We were surprised to observe two cell lines displaying patterns of mosaic CNV calls consistent with "trisomy rescue" of chromosomes 5 (Genea48) or 16 (HUES71) (Figure S4B). These trisomies must have resulted from meiotic nondisjunction, since they have three distinct haplotypes on segments of these chromosomes, as opposed to two imbalanced haplotypes that might arise from mitotic errors. These findings indicate that at least some structural variants we observed were present in human embryos at the time of hESC derivation, which is consistent with their relatively high prevalence in oocytes from older mothers (Hassold et al., 1995). Trisomic cells may be "rescued" to a diploid state by losing one of the excess chromosomes to either restore chromosomal balance or cause uniparental disomy. Our findings suggest that meiotic trisomy rescue may also occur *in vitro*, providing a unique opportunity to explore the biology of a process that cannot be readily studied in primary human tissue or hiPSCs.

Finally, we wondered whether small (<1 Mbp) CNVs might wholly or partially affect genes of likely functional relevance for hESC biology (Tables S3I and S3J). We did not see clear evidence of recurrent small CNVs in hESCs (Figures S4C and S4D) but found that one cell line (WIBR2) carried a small heterozygous deletion encompassing *TP53* and two unrelated cell lines (CSES6 and CSES25), as well as distinct heterozygous deletions encompassing *EP300* that were not observed in human controls (Table S3J). Similar heterozygous deletions at Chr17p13.1 that include *TP53* have been shown to confer growth advantage to hPSCs (Amir et al., 2017). Moreover, the EP300 gene product acetylates and stabilizes p53 (Gayther et al., 2000), suggesting that its reduced dosage could contribute to reduced p53 activity. Overall, our results suggest that certain small-culture-acquired CNVs may functionally impact hPSC biology.

Frequency of SNVs in hESCs

To take advantage of the single-nucleotide resolution that WGS enables, we tested for missense and loss-of-function (LoF) SNVs that can profoundly alter cellular function by affecting both coding regions and functionally important non-coding regions of the genome. Although individual SNVs sufficient to cause human disease are rare in a given individual, in aggregate they affect over 300 million people worldwide (Nguengang Wakap et al., 2020). We considered SNV calls from the autosomes and X chromosomes that were supported by high confidence and high quality (HC-HQ) sequence data using filters similar to those used to analyze whole-genome sequences in the gnomAD v2.1 database (Karczewski et al., 2020; Figure 5A). We observed an average SNV burden per hESC line of 244 LoF variants and 11,483 missense variants, which was indistinguishable with that described in gnomAD whole-genome sequences from humans of diverse ancestries (Figure 5B).

Given that deleterious variants are rare in the general population due to negative selective pressure, we restricted our analysis to variants present at an allele frequency (AF) of less than 0.001 (0.1%) among 60,706 exomes represented in the ExAC database (Lek et al., 2016), which are non-overlapping with these gnomAD whole genomes. To conservatively enrich for likely deleterious variants among these rare SNVs, we used the bioinformatic prediction tools "combined annotation dependent depletion" (CADD; Kircher et al., 2014) and "deleterious annotation of genetic variants using neural networks" (DANN; Quang et al., 2015) to identify the variants predicted to be among the top 1% most deleterious in the human genome (CADD phred >20 and DANN >0.99). When we compared the 9,982 SNVs meeting these criteria in hESC lines with similarly filtered SNVs from whole genomes from gnomAD, we again did not observe an enriched burden of deleterious SNVs in hESCs (Figure 5C). Together, these findings are consistent with the null hypothesis

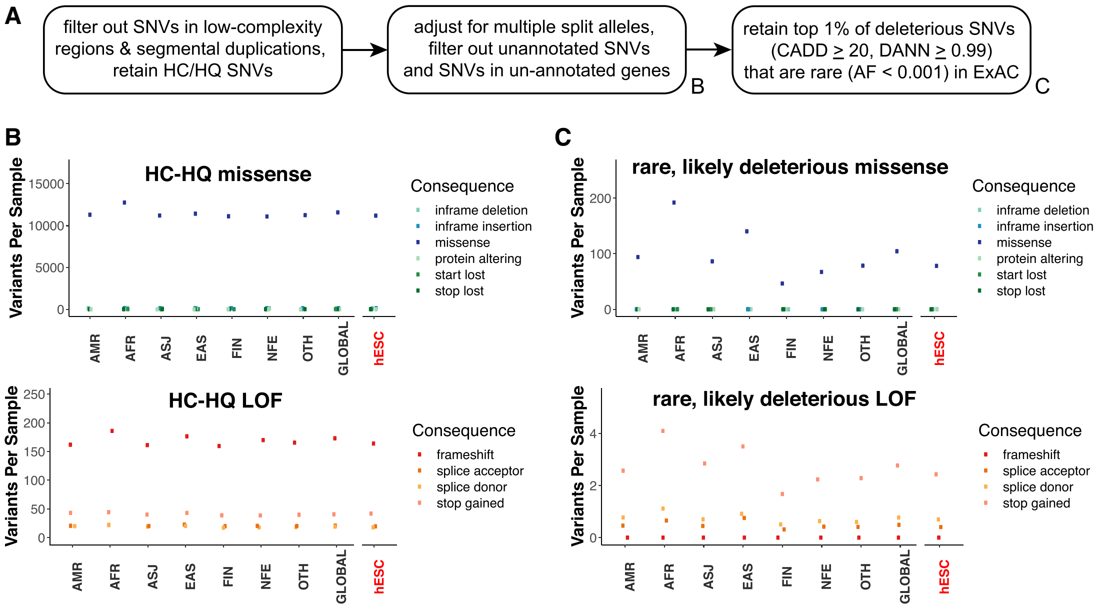

Figure 5. The overall SNV burden in hESCs resembles that of human populations

(A) Workflow for SNV identification and prioritization based on sequencing quality, bioinformatic prediction of deleteriousness, and ExAC allele frequency. HC/HQ, high confidence/high quality.

(B) Per-sample burden of missense and LoF SNVs passing HC/HQ filters across different human ancestries and in the analyzed hESCs.

(C) Per-sample burden of rare and predicted deleterious missense and LoF SNVs.

AMR, Ad-mixed American ancestry; AFR, African ancestry; ASJ, Ashkenazi Jewish ancestry; EAS, East Asian ancestry; FIN, Finnish ancestry; NFE, non-Finnish European ancestry; OTH, other ancestry; GLOBAL, all whole-genome samples in gnomAD; hESC, hESC samples in this study.

that, under the conditions tested, hESCs do not accumulate an excess burden of SNVs that is detectable above the sampling noise of normal inter-individual genetic variation.

Cancer-associated SNVs and structural variants

Genetic variants associated with cancer are of particular interest to the stem cell community since they might alter hESC genomic stability and growth characteristics, disrupt hESC differentiation and cellular phenotypes in differentiated cells, or increase the risk of cancerous growths arising from hESC-derived cells after transplant. We therefore asked whether any of the SNVs observed in hESCs fell within genes having a documented "Tier 1" activity relevant to cancer as annotated in the Catalog of Somatic Mutations in Cancer (COSMIC: https://cancer.sanger.ac.uk/cosmic; Tate et al., 2019). We then asked which of the 382 variants meeting these criteria had been observed in human cancers in COSMIC at least twice (n = 51) and were bioinformatically predicted to be cancer causing by functional analysis through hidden Markov models (FATHMM: http://fathmm.biocompute.org.uk/cancer.html; Shihab et al., 2013). This analysis revealed 14 unique heterozygous missense variants across 10 genes in 15 hESC lines (Table S4A; Figure 6B), including three of five mutations in *TP53* that we had previously identified by exome sequencing (Merkle et al., 2017). Several of the other variants suggested the recurrent involvement of the p53 and DNA damage response pathways and are consistent with our earlier discovery of heterozygous small deletions affecting *TP53* and *EP300*, though the functional role of these variants in hPSCs survival or proliferation is unclear (Figure 6B). Of the genes with COSMIC-associated variants, both *TP53* and *EGFR* were independently found to be recurrently mutated in hESCs (Avior et al., 2021).

Fertility-associated variants

The majority of hESCs we studied here were derived from donated embryos that were excess to the needs of couples seeking assisted reproduction at *in vitro* fertilization (IVF) clinics. We thus considered whether genetic variants affecting fertility would be present among the sequenced hESC lines, since there is considerable interest in differentiation of hPSCs into germ cells for studies of human meiosis and gametogenesis (Sasaki et al., 2015; Zhou et al., 2016). We examined the intersection between a list of genes associated with infertility from the literature (Mallepaly et al., 2017; O'Flynn O'Brien et al., 2010; Venkatesh et al., 2014), and genes in which inactivating heterozygous mutations are under strong negative selective pressure and are therefore likely to cause dominant disease as identified using the loss-of-function observed/expected upper bound fraction (LOEUF) metric from studies of human genome variation (Karczewski et al., 2020). We conservatively set LOEUF to 0.33 or less, revealing variants in 12 fertility-associated genes meeting these criteria. To increase our confidence in the disease relevance of these variants, we further restricted our analysis to genes whose disruption is associated with autosomal dominantly inherited disease in the manually curated clinical genomic database (CGD: https://research.nhgri.nih.gov/CGD/; Solomon et al., 2013) and identified eight variants affecting six of the 12 previously identified genes (Figure 6C; Table S4B). These genes are associated with infertility due to autosomal dominant hypogonadotropic hypogonadism, premature ovarian failure, or fetal growth and

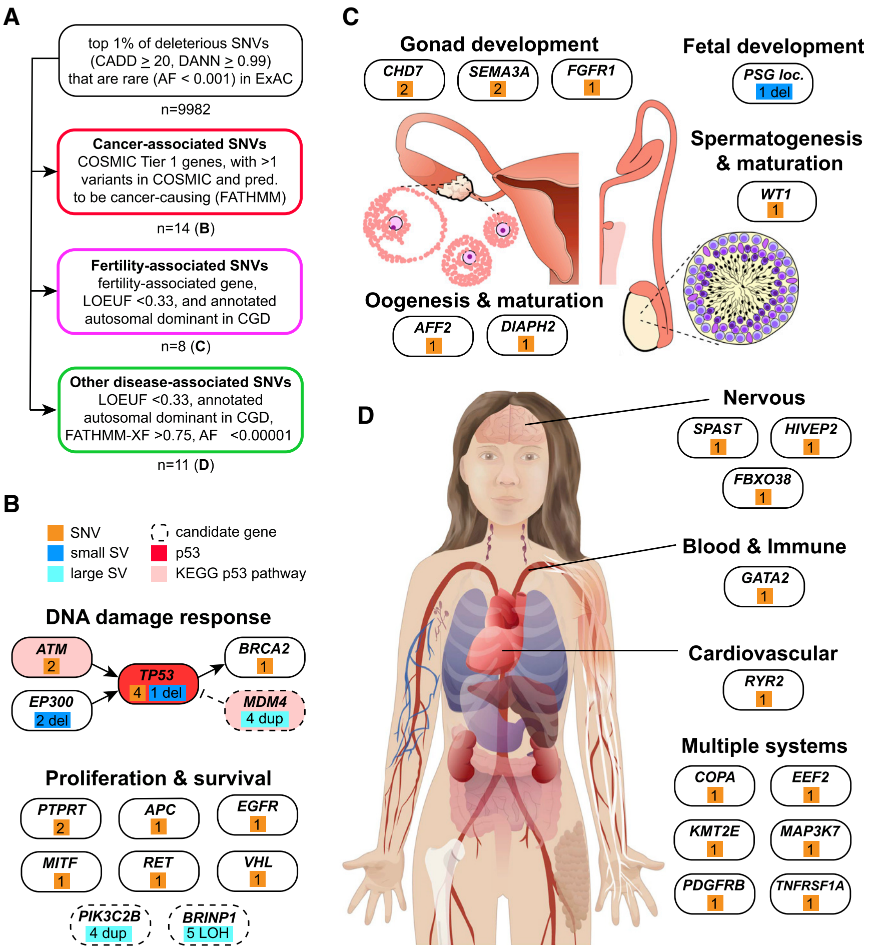

Figure 6. Genes and systems affected by likely deleterious SNVs in hESCs

(A) Schematic of approach used to identify disease-associated genetic variants in hESCs, and number of variants passing these filters.

(B) Analysis of cancer-associated variants suggests broader involvement of the p53 pathway (Kyoto Encyclopedia of Genes and Genomes, KEGG, red shading).

(C) Numerous fertility-associated genes carried deleterious mutations, suggesting potential causes of sub-fertility in some of the couples who donated embryos for the hESC derivation.

(D) SNVs in genes associated with autosomal dominant disease affect multiple body systems *in vivo* and would likely affect cell types generated from hPSCs. See also Table S4.

survival, raising the intriguing possibility that these variants might explain the cause of sub-fertility in couples who donated their embryos to generate the hESC lines we analyzed.

Disease-associated SNVs

Finally, we screened for variants that might cause disease-relevant phenotypes *in vitro* or compromise the safety of hESC-based regenerative medicine. As above, we focused on genes with LOEUF scores below 0.33 (n = 2000) and associated with autosomal dominant human disease in CGD (n = 324). To restrict ourselves to the variants in autosomal genes most likely to be pathogenic, we considered those bioinformatically predicted to be pathogenic by FATHMM-XF (score >0.75, n = 146) (Rogers et al., 2018) and DANN (score >0.999, n = 36) and present at an allele frequency of less than 1×10^{-5} in ExAC (n = 11). These variants (Figure 6D; Table S4C) affected genes required for normal development and might therefore interfere with the generation of specific cell types. For example, one cell line (KCL019) carried a variant in *GATA2*, a transcription factor important for immune cell development and associated with immunodeficiency and leukemia when disrupted (Collin et al., 2015). Each of the SNVs described above were manually verified using existing whole exome sequencing (WES) data (Table S4D), and users can review aligned sequence reads for other variants of interest, as described below.

Culture-acquired genetic variants

Many large CNVs are selected for during the culture of hPSCs (Draper et al., 2004), and previous analysis of whole-exome sequencing data revealed SNVs with allele-specific bias in sequencing depth that were likely to be culture acquired (Merkle et al., 2017; Table S4E). To extend these analyses, we exploited the discovery of many genetic variant classes called from WGS analysis to ask which of them might be culture acquired in hESCs. We considered all large CNVs, since they are rare in human populations but restricted our analysis to "singleton" small CNVs and SNVs present just once among genetically unrelated cell lines to enrich for variants more likely to be culture acquired and then further classified SNVs as potentially deleterious, potentially cancer associated, or known cancer associated (Table S4F). We then used linear regression to correlate the abundance of these genetic variants with candidate variables and confirmed and extended previous reports indicating that the number of large CNVs per cell line is significantly (p = 0.03) correlated with the passage number at the time of sequencing (Halliwell et al., 2020; Figure S5; Tables S4F and S4G). However, the only other significant associations we detected were with ancestry, suggesting that in the present experimental design, any signal from culture-acquired genetic variants is swamped by the magnitude of inherited genetic variation present among analyzed cell lines. Overall, these findings support our interpretation that hESC genomes largely resemble those of human populations and are therefore powerful tools for studying human biology.

Tools for rational hESC line selection

The breadth of findings that can be garnered from WGS data raised the question of how genomic information can best be harnessed by stem cell biologists to rationally select an appropriate cell line for a particular application. Which variants are likely benign, and which might limit the utility of a cell line in a

A

Cell line	Fixed large structural variant	Mosaic large structural variant	Cancer-associated variant	Fertility-associated variant	Diease-associated variant	PRS outliers (>2 SD)	Passages thaw to bank	Passage at sequencing	Genotyped sex	Banked?	GMP-prepared (G)?
CHB04						4	2	22	M	Y	
CHB05		1			1	1	3	24	F	Y	
CHB06							3	24	F	Y	
CHB08						1	3	25	F	Y	
CHB09						1	3	23	M	Y	
CHB10						2	2	25	M	Y	
CHB11			1			3	3	37	F	Y	
CHB12				1			3	26	F	Y	
CSES02		2				3	3	15	F	Y	
CSES04						2	4	14	M	Y	
CSES06		1		1			4	26	F	Y	
CSES07		1				1	3	26	F	Y	
CSES12		1					4	18	M	Y	
CSES15	1					3	3	27	M	Y	
CSES25		1	1				2	14	F	Y	
CT1						2	3	40	F	Y	
CT2			1				3	11	F	T	
CT3			1			1	3	17	F	Y	
CT4						3	2	15	F	Y	
Elf1						3	4	12	F	Y	
ESI-017		1		1		2	3	31	F	Y	
ESI-035		1	1			1	3	36	F	Y	
ESI-049						2	3	33	M	Y	
ESI-051	1						2	35	F	Y	
ESI-053						1	2	28	F	Y	
Genea002				1		2	3	17	M	Y	
Genea015						1	2	19	M	Y	
Genea016						3	2	20	F	Y	
Genea042						2	2	19	F	Y	
Genea043		2				1	2	12	M	Y	
Genea047						2	2	10	F	Y	
Genea048		1		1			2	8	M	Y	
Genea052	1					3	2	11	M	Y	
Genea057							3	13	F	Y	
HS346						4	3	25	F	Y	
HS401		1				2	3	25	M	Y	
HS420						1	3	24	M	Y	
HS975						3		8	F		G
HS980						2		8	F		G
HS983a								7	M		G
HS999		1				1		12	F		G
HS1001						2		14	F		G
HUES42			1			1	3	22	M	Y	
HUES44			1			3	3	16	F	Y	
HUES45					1	1	4	21	F	Y	
HUES48		1				2	2	23	F	Y	
HUES49							3	25	F	Y	
HUES53			1			1	3	16	M	Y	
HUES62				1		2	4	18	F	Y	
HUES63						2	3	15	M	Y	
HUES64						2	2	14	M	Y	
HUES65				1		1	4	15	M	Y	
HUES66		1				1	3	15	F	Y	
HUES68		2					4	27	F	Y	
HUES69					2	2	4	21	F	Y	
HUES70						2	4	27	F	Y	
HUES71		2				1	3	28	F		
HUES72		1	1			1	3	20	F	Y	
HUES73		3				3	3	18	M		
HUES74						2	4	7	M	Y	
HUES75						1	4	7	F	Y	
I3						1	3	27	F		
I4						2	3	29	F		
KCL019					1	2		8	F		G
KCL020				1		1		30	M		G
KCL022		2			1			22	M		G
KCL031						4		17	M		G
KCL032	1				1			7	F		G
KCL033	1					1		17	F		G
KCL034						1		17	M		G
KCL037						3		7	M		G
KCL038								9	M		G
KCL039					1			8	M		G
KCL040						2		22	F		G
Man11						1		27	F		G
Man12						1		24	M		G
MEL1						1	2	20	M	Y	
MEL2		1				1	3	28	F	Y	
MEL3		2				1	3	38	F	Y	
MEL4						4	3	27	F	Y	
MShef02				1		1		14	F		G
MShef03		2				2		22	F		G
MShef04								18	M		G
MShef05								16	M		G
MShef07		1				1		16	M		G
MShef08								13	F		G
MShef10			1					18	M		G
MShef11						1		10	M		G
MShef12						2		35	F		G
MShef13						1		8	M		G
MShef14						3		13	F		G
RUES1		1				2	3	25	M	Y	
RUES2		2				1	6	18	F	Y	
Shef3.2						3	3	13	M	Y	
Shef6.1		3				2	2	12	F	Y	
UCLA1						1	2	15	F	Y	
UCLA2						1	2	14	M	Y	
UCLA3						2	2	16	F	Y	
UCLA4					1	2	2	16	F	Y	
UCLA5						1	2	26	F	Y	
UCLA6							2	20	M	Y	
UCLA7		1				2	2	21	F	Y	
UCLA8						1	2	15	F	Y	
UCLA9							2	16	F	Y	
UCLA10						1	2	15	M	Y	
UCLA11						2	3	16	M	Y	
UCLA12		1				2	2	16	F	Y	
UCLA13				1		1	2	16	M	Y	
UCLA14							2	20	F	Y	
UCLA15		1				3	3	19	F	Y	
UCLA16						2	2	16	F	Y	
UCLA17	1		1			4	3	18	F	Y	
UCLA18		1					2	26	F	Y	
UCSF4						1	3	19	F	Y	
UM4-6						2	2	15	M	Y	
UM14-1			1			1	2	26	M	Y	
UM22-2						1	2	26	F	Y	
UM33-4						2	2	25	F	Y	
UM77-2			1				2	13	F	Y	
UM78-2	1					3	2	13	M	Y	
UM121-7		1	1			2	2	10	M	Y	
WA01 (H1)		1				2	2	23	M	Y	
WA07 (H7)						1	2	15	F	Y	
WA09 (H9)						1	2	33	F	Y	
WA13 (H13)		1				1	2	26	M	Y	
WA14 (H14)						2	2	33	M	Y	
WA15	1					2	2	21	M	Y	
WA17						5	3	31	M	Y	
WA18						2	2	12	M	Y	
WA19					1	3	2	9	M	Y	
WA20	3	1				2	2	11	F	Y	
WA21	1		1			1	2	23	M	Y	
WA22						3	2	28	F	Y	
WA23		3				1	2	14	M	Y	
WA24					1		2	20	M	Y	
WA25							2	15	F	Y	
WA26							2	14	F	Y	
WA27		1					2	15	F	Y	
WIBR1*	3						2	34	M	Y	
WIBR2*						1	3	33	F	Y	
WIBR3*							3	23	F	Y	
WIBR5*						1	3	13	F	Y	
WIBR6*	1					1	3	11	M	Y	

B

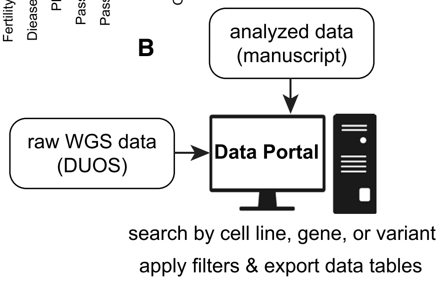

C

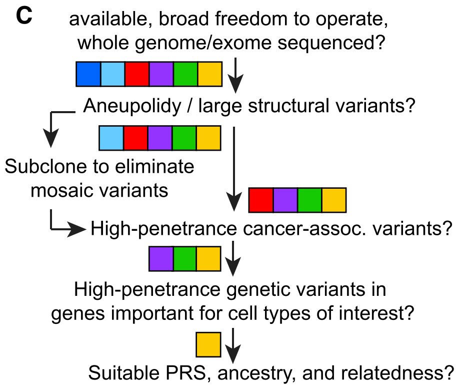

(legend on next page)

given application? To help the community address these questions, we generated three complementary resources. First, we summarize some of the most relevant results presented in this study in the form of a convenient lookup table (Figure 7A) and provide annotated tables of the variants we identified (Tables S1–S4). Second, the raw sequencing data are freely available to interested groups via a controlled-access database (DUOS: https://duos.broadinstitute.org/, dataset DUOS-000121). Finally, we have created a user-friendly online data portal (https://hscgp.broadinstitute.org/hscgp) (Figure 7B) that enables users with more limited computational expertise to readily search for sequence variants of interest among sequenced hESC lines. For example, a search for *TP53* reveals all variants in the gene that were detected in the sequenced cell lines, the names of those cell lines, as well as bioinformatic predictions about the likely consequences of these variants. Search results can be graphically visualized and exported for further analysis in a variety of formats. Specific cell lines can also be interrogated for the presence of variants of interest, and raw sequencing alignments can be visualized via the integrative genomics viewer IGV (Robinson et al., 2011).

DISCUSSION

Techniques commonly used to evaluate the genetic integrity of hESCs, including high-density SNP microarrays and karyotyping by G-banding, have limited spatial resolution and limited power to detect mosaic events (Baker et al., 2016). Here, we show that high-coverage (>25×) WGS enables the robust identification of potentially relevant structural and SNVs, including thousands of small CNVs that are not detectable using traditional methods and several of which are likely of functional relevance. As the price of WGS is steadily dropping and provides considerably more information on genetic variation than many other methods, our experience suggests that WGS may gradually become the tool of choice for the genetic analysis of both hESCs and hiPSCs. In particular, WGS may prove valuable in the selection of hPSCs for use in disease modeling and gene editing experiments, as well as in the interpretation of data arising from these models and retrospective multimodal analysis, as many of these cell lines included in this study have also been used to generate complementary datasets such as RNA-seq and DNA methylation.

The origin and impact of structural genetic variants

We found that almost a third of hESC lines in this study carried large structural variants, of which approximately half were mosaic (Figures 4J, 4K, and 7A; Table S3B). Mosaic variants might arise in culture and confer selective advantage to affected cells, leading to the expansion and eventual fixation of the variant. Indeed, of the nine hESC lines carrying advantageous duplications at Chr20q11.21, six were mosaic and three were fixed (Figure 4B). Alternatively, mosaicism could arise from negative selective pressures, since approximately half of all preimplantation human embryos carry large structural genetic variants (Fragouli et al., 2019), and we observed at least two instances of apparent trisomy rescue (Figure S3H). Fixed structural and sequence variants might have arisen in culture and reached fixation or might be inherited, as seen in the sibling cell lines KCL032 and KCL033 that carried identical large duplications on Chr5 (Table S3B), or cell lines carrying SNVs associated with disease or sub-fertility (Figure 6).

In addition to suggesting mechanisms explaining the frequent recurrence of known duplications at Chr20q11.21 (Figure 4H) and Chr1q32.1 (Figure 4L), we discovered a recurrent CN-LOH event on Chr9q (Figure 4L) that would alter the expression levels of imprinted or differentially methylated genes that regulate survival or proliferation. Indeed, Chr9q LOH is frequently observed in certain types of cancer (Hirao et al., 2005; Yizhak et al., 2019; Jakubek et al., 2020), and the gene *BRINP1* (a.k.a. *DBCCR1* or *DBC1*) at Chr9q33.1 as well as the microRNA miR-181a2/181b2 at Chr9q33.3 are frequently deleted or hypermethylated in cancer (Izumi et al., 2005; Mei et al., 2017). Future transcriptional and epigenetic profiling studies of hPSCs may reveal specific genetic variants associated with the recurrence of this recurrent CN-LOH variant.

Suggestions for rational hPSC selection

The unique constellation of inherited and acquired genetic variants present across the studied cell lines raises the question of how knowledge of these variants can rationally inform which lines should be selected. For the purpose of regenerative medicine, where safety should be considered, polyploid cells have been knowingly transplanted into humans without apparent ill effect (Nelson et al., 2002), and while we feel that most groups would agree that a cell line carrying a *TP53* mutation conferring a lifetime cancer risk of nearly 100% should not be transplanted into patients if a suitable alternative exists, other variants should be considered on a case-by-case basis depending on research needs. In contrast, for the purpose of basic research, the presence of potentially damaging variants associated with fertility or other disease may be of interest to groups studying human development and modeling the associated diseases. Furthermore, since many genes are expressed in a cell-type-dependent manner, even genetic variants predicted to be highly deleterious in one cell type may be unproblematic for applications that focus on another cell type. We therefore suggest a scheme (Figure 7C) that can be adapted to achieve the appropriate balance of risk and benefit for a particular application. First, we reason that most groups would prefer to work with cell lines having minimal restrictions on the freedom to use or share the lines and their derivatives, and whose genomic structure has been extensively characterized. Second, we suggest avoiding lines with aneuploidy or large structural variants, although it might be possible

Figure 7. Genetically informed rational cell line selection

(A) Graphical summary of the number of large fixed and mosaic structural variants (dark and light blue, respectively), SNVs likely associated with cancer (red), fertility (magenta), or other diseases (green), "outlier" PRS, and summary data for each analyzed hESC line. *please see note about WIBR lines in Tables S1A, S2, S3, and S4 for details about variants.

(B) Types of data availability for this resource.

(C) Suggested workflow for rational hPSC line selection based on genetic information.

CellPress
OPEN ACCESS

to "rescue" lines with mosaic variants by sub-cloning or by requesting an earlier passage line that may lack the variant. Third, most groups would avoid cell lines carrying cancer- and disease-associated variants, though we note that other groups may choose to exploit these variants. Fourth, the PRS for a trait of interest, ancestry, and relatedness of the cell line may reveal cell lines are most likely to display desired traits.

Perspective

Together, our analysis demonstrates that the overall numbers of small CNVs and SNVs identified in hESCs resemble those of somatic cells from a similarly scaled population of donors, demonstrating the relative stability of their genomes and validating hESCs as a powerful tool to study human development and disease and as a useful source of clinically important cell populations. Indeed, the vast majority of variants we observed are of unknown significance, and even variants that have been associated with disease rarely have strong evidence demonstrating that they play a causal role. We anticipate that the data provided here will become increasingly valuable as our understanding of genotype-phenotype relationships steadily improves. By providing a searchable online data portal enabling individuals with any level of computational expertise to make use of the resource we report here, we hope that the reproducibility of research findings from hPSC studies and their ultimate use in clinical applications will be improved.

Limitations of the study

While we strove to identify the most relevant genetic variants present in hESC lines, our analyses were not exhaustive. We did not consider inversions, translocations, repetitive genomic regions, mitochondrial DNA sequences, epigenetic differences, or variants on the Y chromosome. While the WGS data used in this study provides an unprecedented view of stem cell genomes, its short read length (150-base-paired-end reads), coupled with the inherent variation in sequencing read depth due to DNA replication, does have some limitations that might be mitigated in part by future studies using long-read sequencing technologies. For instance, our analysis was limited to variants $\lesssim$50 bp or $\gtrsim$1.1 kbp, and our analysis of structural variant mosaicism was limited to variants $\gtrsim$1 Mbp. Though the tagmentation-based methods, we used in library preparation did not appear to result in any sequence bias in CNV calls made by DOC analysis (Figure S3D), we cannot formally exclude this possibility. Similarly, it is possible that variable sequencing depth due to replication timing and sequencing bias might result in erroneous CNV calls. While IRS testing validated the vast majority of these small variants, error rates were highest among duplications under 20 kbp. We encourage groups to independently verify variants of potential biological significance, such as deletions affecting haploinsufficient or cancer-associated genes (Table S3J). All 33 SNVs highlighted in this text were manually confirmed by reviewing WES data from these same cell lines as well as IGV traces accessible via the data portal (https://hscgp.broadinstitute.org/hscgp). However, the accuracy of HLA haplotype estimation is constrained by the limited number of informative SNPs (De Bakker et al., 2006), and haplotypes should be verified prior to use in any downstream application. We sought to identify sequence variants likely to affect the function of hPSCs or their derivatives using a combination of gene-level and variant-level filters based on manually curated databases and bioinformatic prediction algorithms and note that databases are not comprehensive and prediction algorithms are imperfect, making it difficult to predict which variants are sufficient to cause disease. Conversely, our stringent bioinformatic selection criteria likely exclude some functionally relevant variants. For example, filtering on LOEUF excluded all variants in *TP53* and a D90A variant in *SOD1* associated with incompletely penetrant amyotrophic lateral sclerosis (Al-Chalabi et al., 1998) that may be relevant to groups modeling neurodegenerative disease, and our sequencing quality filters removed two of the five variants previously identified in *TP53* since they were present at low allelic fractions. We therefore encourage groups to interrogate the full dataset for relevant variants in their cell line(s) of interest, using the web resource provided or by re-analyzing the raw data (DUOS: https://duos.broadinstitute.org/, dataset DUOS-000121). We also anticipate that groups may wish to utilize the datasets presented here in combination with transcriptomic data to map expression quantitative trait loci (eQTL) in hESCs or their differentiated progeny.

STAR★METHODS

Detailed methods are provided in the online version of this paper and include the following:

- KEY RESOURCES TABLE
- RESOURCE AVAILABILITY
  - Lead contact
  - Materials availability
  - Data and code availability
- EXPERIMENTAL MODEL AND SUBJECT DETAILS
- METHOD DETAILS
  - Whole genome sequencing and genotyping
  - Ancestry, relatedness, and HLA analysis
  - Polygenic risk score (PRS) computations
  - CNV calling from read depth variation
  - Identification of large structural variants
  - Validation of large structural variants
  - Detection and interpretation of small CNVs
  - Identification of SNVs in hESCs
  - Data portal architecture and implementation
  - SNV characterization and prioritization
- QUANTIFICATION AND STATISTICAL ANALYSIS

SUPPLEMENTAL INFORMATION

Supplemental information can be found online at https://doi.org/10.1016/j.stem.2022.01.011.

ACKNOWLEDGMENTS

We thank Maura Charlton, Genevieve Saphier, Diane Santos, Melissa Smith, Kristen Elwell, Mary Anna Yram, Stacey Ellender, Liz Bevilacqua, Diane Gage, and Anna Neumann for their assistance with acquiring hESC lines, coordinating the sequencing and genotyping workflows, and preparing data for submission to data repositories. At the Broad Institute of MIT and Harvard, the Genomics Platform performed sample preparation, sequencing, and data storage; and Jonathan Bistline, Andrew Zimmer, and the KDUX team of the Data Sciences Platform helped develop web tools. We are also grateful

for the many institutions from around the world who generously provided their cell lines and supported the publication of the results, who are listed in full in Table S1. Costs associated with acquiring and sequencing hESC lines were supported by the Howard Hughes Medical Institute and the Stanley Center for Psychiatric Research at the Broad Institute of MIT and Harvard. F.T.M. is a New York Stem Cell Foundation, Robertson Investigator (NYSCF-R-156) and was supported by the Medical Research Council (MR/P501967/1), Academy of Medical Sciences (SBF001\1016), Wellcome Trust and Royal Society (211221/Z/18/Z), the Chan Zuckerberg Initiative (191942), and the NIH (5K99NS083713).

AUTHOR CONTRIBUTIONS

F.T.M., S.G., S.A.M., and K.E. conceptualized, supervised, and jointly administered the project. Formal analysis, curation, and visualization of data was performed by F.T.M. as well as S.G., G.G., R.E.H., S.K., D.M., and C.O. Development of methodologies to analyze and validate data was carried out by G.G., R.E.H., S.K., and K.J.K., under the supervision of S.A.M. and D.G.M. D.M. helped to develop software and organize resources for hosting and visualizing WGS data. C.P. and M.P. provided access to unpublished WGS data from individuals of Latin American and African American ancestry unaffected by schizophrenia for CNV analysis. Funding for the study was acquired by S.A.M. and K.E. The original draft of the manuscript was written by F.T.M., with contributions from S.G., G.G., R.E.H., S.K., and D.M. The manuscript was reviewed and edited by F.T.M., S.G., S.A.M., and K.E., and all authors read and approved the manuscript.

DECLARATION OF INTERESTS

K.E. is a founder of Q-State Biosciences, Quralis, and Enclear Therapies; an employee and shareholder of BioMarin; and a member of *Cell Stem Cell*'s advisory board. K.J.K. is a consultant for Vor Biopharma, and C.O. is now an employee of 54gene.

INCLUSION AND DIVERSITY

We worked to ensure diversity in experimental samples through the selection of the cell lines. We worked to ensure diversity in experimental samples through the selection of the genomic datasets. One or more of the authors of this paper self-identifies as an underrepresented ethnic minority in science. While citing references scientifically relevant for this work, we also actively worked to promote gender balance in our reference list.

Received: April 10, 2020
Revised: October 29, 2021
Accepted: January 24, 2022
Published: February 16, 2022

REFERENCES

1000 Genomes Project Consortium, Abecasis, G.R., Auton, A., Brooks, L.D., DePristo, M.A., Durbin, R.M., Handsaker, R.E., Kang, H.M., Marth, G.T., and McVean, G.A. (2012). An integrated map of genetic variation from 1,092 human genomes. Nature *491*, 56–65.

Al-Chalabi, A., Andersen, P.M., Chioza, B., Shaw, C., Sham, P.C., Robberecht, W., Matthijs, G., Camu, W., Marklund, S.L., Forsgren, L., et al. (1998). Recessive amyotrophic lateral sclerosis families with the D90A SOD1 mutation share a common founder: evidence for a linked protective factor. Hum. Mol. Genet. *7*, 2045–2050.

Altemose, N., Miga, K.H., Maggioni, M., and Willard, H.F. (2014). Genomic characterization of large heterochromatic gaps in the human genome assembly. PLoS Comput. Biol. *10*, e1003628.

Amir, H., Touboul, T., Sabatini, K., Chhabra, D., Garitaonandia, I., Loring, J.F., Morey, R., and Laurent, L.C. (2017). Spontaneous single-copy loss of TP53 in human embryonic stem cells markedly increases cell proliferation and survival. Stem Cells *35*, 872–885.

Avery, S., Hirst, A.J., Baker, D., Lim, C.Y., Alagaratnam, S., Skotheim, R.I., Lothe, R.A., Pera, M.F., Colman, A., Robson, P., et al. (2013). BCL-XL mediates the strong selective advantage of a 20q11.21 amplification commonly found in human embryonic stem cell cultures. Stem Cell Rep. *1*, 379–386.

Avior, Y., Lezmi, E., Eggan, K., and Benvenisty, N. (2021). Cancer-related mutations identified in primed human pluripotent stem cells. Cell Stem Cell *28*, 10–11.

Avior, Y., Sagi, I., and Benvenisty, N. (2016). Pluripotent stem cells in disease modelling and drug discovery. Nat. Rev. Mol. Cell Biol. *17*, 170–182.

Baker, D., Hirst, A.J., Gokhale, P.J., Juarez, M.A., Williams, S., Wheeler, M., Bean, K., Allison, T.F., Moore, H.D., Andrews, P.W., and Barbaric, I. (2016). Detecting genetic mosaicism in cultures of human pluripotent stem cells. Stem Cell Rep. *7*, 998–1012.

Bulik-Sullivan, B., Finucane, H.K., Anttila, V., Gusev, A., Day, F.R., Loh, P.-R., Duncan, L., et al.; ReproGen Consortium; Psychiatric Genomics Consortium; Genetic Consortium for Anorexia Nervosa of the Wellcome Trust Case Control Consortium 3 (2015). An atlas of genetic correlations across human diseases and traits. Nat. Genet. *47*, 1236–1241.

Canham, M.A., Van Deusen, A., Brison, D.R., De Sousa, P.A., Downie, J., Devito, L., Hewitt, Z.A., Ilic, D., Kimber, S.J., Moore, H.D., et al. (2015). The molecular karyotype of 25 clinical-grade human embryonic stem cell lines. Sci. Rep. *5*, 17258.

Chang, C.C., Chow, C.C., Tellier, L.C.A.M., Vattikuti, S., Purcell, S.M., and Lee, J.J. (2015). Second-generation PLINK: rising to the challenge of larger and richer datasets. GigaScience *4*, 7.

Chen, A.E., Egli, D., Niakan, K., Deng, J., Akutsu, H., Yamaki, M., Cowan, C., Fitz-Gerald, C., Zhang, K., Melton, D.A., and Eggan, K. (2009). Optimal timing of inner cell mass isolation increases the efficiency of human embryonic stem cell derivation and allows generation of sibling cell lines. Cell Stem Cell *4*, 103–106.

Chiang, C., Scott, A.J., Davis, J.R., Tsang, E.K., Li, X., Kim, Y., Hadzic, T., Damani, F.N., Ganel, L., et al.; GTEx Consortium (2017). The impact of structural variation on human gene expression. Nat. Genet. *49*, 692–699.

Collin, M., Dickinson, R., and Bigley, V. (2015). Haematopoietic and immune defects associated with GATA2 mutation. Br. J. Haematol. *169*, 173–187.

Corder, E.H., Saunders, A.M., Strittmatter, W.J., Schmechel, D.E., Gaskell, P.C., Small, G.W., Roses, A.D., Haines, J.L., and Pericak-Vance, M.A. (1993). Gene dose of apolipoprotein E type 4 allele and the risk of Alzheimer's disease in late onset families. Science *261*, 921–923.

De Bakker, P.I.W., McVean, G., Sabeti, P.C., Miretti, M.M., Green, T., Marchini, J., Ke, X., Monsuur, A.J., Whittaker, P., Delgado, M., et al. (2006). A high-resolution HLA and SNP haplotype map for disease association studies in the extended human MHC. Nat. Genet. *38*, 1166–1172.

Dean, M., Carrington, M., Winkler, C., Huttley, G.A., Smith, M.W., Allikmets, R., Goedert, J.J., Buchbinder, S.P., Vittinghoff, E., Gomperts, E., et al. (1996). Genetic restriction of HIV-1 infection and progression to AIDS by a deletion allele of the CKR5 structural gene. Science *273*, 1856–1862.

Ding, Q., Edwards, M.M., Wang, N., Zhu, X., Bracci, A.N., Hulke, M.L., Hu, Y., Tong, Y., Hsiao, J., Charvet, C.J., et al. (2021). The genetic architecture of DNA replication timing in human pluripotent stem cells. Nat. Commun. *12*, 6746.

Draper, J.S., Smith, K., Gokhale, P., Moore, H.D., Maltby, E., Johnson, J., Meisner, L., Zwaka, T.P., Thomson, J.A., and Andrews, P.W. (2004). Recurrent gain of chromosomes 17q and 12 in cultured human embryonic stem cells. Nat. Biotechnol. *22*, 53–54.

Fragouli, E., Munne, S., and Wells, D. (2019). The cytogenetic constitution of human blastocysts: insights from comprehensive chromosome screening strategies. Hum. Reprod. Update *25*, 15–33.

Francoz, S., Froment, P., Bogaerts, S., De Clercq, S., Maetens, M., Doumont, G., Bellefroid, E., and Marine, J.C. (2006). Mdm4 and Mdm2 cooperate to inhibit p53 activity in proliferating and quiescent cells in vivo. Proc. Natl. Acad. Sci. USA *103*, 3232–3237.

Gayther, S.A., Batley, S.J., Linger, L., Bannister, A., Thorpe, K., Chin, S.F., Daigo, Y., Russell, P., Wilson, A., Sowter, H.M., et al. (2000). Mutations truncating the EP300 acetylase in human cancers. Nat. Genet. *24*, 300–303.

CellPress
OPEN ACCESS

Guerreiro, R., Wojtas, A., Bras, J., Carrasquillo, M., Rogaeva, E., Majounie, E., Cruchaga, C., Sassi, C., Kauwe, J.S.K., Younkin, S., et al. (2013). *TREM2* variants in Alzheimer's disease. N. Engl. J. Med. *368*, 117–127.

Halliwell, J., Barbaric, I., and Andrews, P.W. (2020). Acquired genetic changes in human pluripotent stem cells: origins and consequences. Nat. Rev. Mol. Cell Biol. *21*, 715–728.

Halliwell, J.A., Baker, D., Judge, K., Quail, M.A., Oliver, K., Betteridge, E., Skelton, J., Andrews, P.W., and Barbaric, I. (2021). Nanopore sequencing indicates that tandem amplification of chromosome 20q11.21 in human pluripotent stem cells is driven by break-induced replication. Stem Cells Dev. *30*, 578–586.

Handsaker, R.E., Van Doren, V., Berman, J.R., Genovese, G., Kashin, S., Boettger, L.M., and McCarroll, S.A. (2015). Large multiallelic copy number variations in humans. Nat. Genet. *47*, 296–303.

Hassold, T., Merrill, M., Adkins, K., Freeman, S., and Sherman, S. (1995). Recombination and maternal age-dependent nondisjunction: molecular studies of trisomy 16. Am. J. Hum. Genet. *57*, 867–874.

Henrichsen, C.N., Chaignat, E., and Reymond, A. (2009). Copy number variants, diseases and gene expression. Hum. Mol. Genet. *18*, R1–R8.

Hirao, S., Hirao, T., Marsit, C.J., Hirao, Y., Schned, A., Devi-Ashok, T., Nelson, H.H., Andrew, A., Karagas, M.R., and Kelsey, K.T. (2005). Loss of heterozygosity on chromosome 9q and p53 alterations in human bladder cancer. Cancer *104*, 1918–1923.

International Schizophrenia Consortium, Purcell, S.M., Wray, N.R., Stone, J.L., Visscher, P.M., O'Donovan, M.C., Sullivan, P.F., and Sklar, P. (2009). Common polygenic variation contributes to risk of schizophrenia and bipolar disorder. Nature *460*, 748–752.

International Stem Cell Initiative, Amps, K., Andrews, P.W., Anyfantis, G., Armstrong, L., Avery, S., Baharvand, H., Baker, J., Baker, D., Munoz, M.B., et al. (2011). Screening ethnically diverse human embryonic stem cells identifies a chromosome 20 minimal amplicon conferring growth advantage. Nat. Biotechnol. *29*, 1132–1144.

Izumi, H., Inoue, J., Yokoi, S., Hosoda, H., Shibata, T., Sunamori, M., Hirohashi, S., Inazawa, J., and Imoto, I. (2005). Frequent silencing of DBC1 is by genetic or epigenetic mechanisms in non-small cell lung cancers. Hum. Mol. Genet. *14*, 997–1007.

Jakubek, Y.A., Chang, K., Sivakumar, S., Yu, Y., Giordano, M.R., Fowler, J., Huff, C.D., Kadara, H., Vilar, E., and Scheet, P. (2020). Large-scale analysis of acquired chromosomal alterations in non-tumor samples from patients with cancer. Nat. Biotechnol. *38*, 90–96.

Jonsson, T., Stefansson, H., Steinberg, S., Jonsdottir, I., Jonsson, P.V., Snaedal, J., Bjornsson, S., Huttenlocher, J., Levey, A.I., Lah, J.J., et al. (2013). Variant of TREM2 associated with the risk of Alzheimer's disease. N. Engl. J. Med. *368*, 107–116.

Kanehisa, M., and Goto, S. (2000). KEGG: kyoto encyclopedia of genes and genomes. Nucleic Acids Res. *28*, 27–30.

Karczewski, K.J., Francioli, L.C., Tiao, G., Cummings, B.B., Alföldi, J., Wang, Q., Collins, R.L., Laricchia, K.M., Ganna, A., Birnbaum, D.P., et al. (2020). The mutational constraint spectrum quantified from variation in 141,456 humans. Nature *581*, 434–443.

Khera, A.V., Chaffin, M., Aragam, K.G., Haas, M.E., Roselli, C., Choi, S.H., Natarajan, P., Lander, E.S., Lubitz, S.A., Ellinor, P.T., and Kathiresan, S. (2018). Genome-wide polygenic scores for common diseases identify individuals with risk equivalent to monogenic mutations. Nat. Genet. *50*, 1219–1224.

Kilpinen, H., Goncalves, A., Leha, A., Afzal, V., Alasoo, K., Ashford, S., Bala, S., Bensaddek, D., Casale, F.P., Culley, O.J., et al. (2017). Common genetic variation drives molecular heterogeneity in human iPSCs. Nature *546*, 370–375.

Kircher, M., Witten, D.M., Jain, P., O'roak, B.J., Cooper, G.M., and Shendure, J. (2014). A general framework for estimating the relative pathogenicity of human genetic variants. Nat. Genet. *46*, 310–315.

Kiskinis, E., Sandoe, J., Williams, L.A., Boulting, G.L., Moccia, R., Wainger, B.J., Han, S., Peng, T., Thams, S., Mikkilineni, S., et al. (2014). Pathways disrupted in human ALS motor neurons identified through genetic correction of mutant SOD1. Cell Stem Cell *14*, 781–795.

Kobold, S., Guhr, A., Kurtz, A., and Löser, P. (2015). Human embryonic and induced pluripotent stem cell research trends: complementation and diversification of the field. Stem Cell Rep. *4*, 914–925.

Landrum, M.J., Lee, J.M., Riley, G.R., Jang, W., Rubinstein, W.S., Church, D.M., and Maglott, D.R. (2014). ClinVar: public archive of relationships among sequence variation and human phenotype. Nucleic Acids Res. *42*, D980–D985.

Laurent, L.C., Ulitsky, I., Slavin, I., Tran, H., Schork, A., Morey, R., Lynch, C., Harness, J.V., Lee, S., Barrero, M.J., et al. (2011). Dynamic changes in the copy number of pluripotency and cell proliferation genes in human ESCs and iPSCs during reprogramming and time in culture. Cell Stem Cell *8*, 106–118.

Lefort, N., Feyeux, M., Bas, C., Féraud, O., Bennaceur-Griscelli, A., Tachdjian, G., Peschanski, M., and Perrier, A.L. (2008). Human embryonic stem cells reveal recurrent genomic instability at 20q11.21. Nat. Biotechnol. *26*, 1364–1366.

Lek, M., Karczewski, K.J., Minikel, E.V., Samocha, K.E., Banks, E., Fennell, T., O'Donnell-Luria, A.H., Ware, J.S., Hill, A.J., Cummings, B.B., et al. (2016). Analysis of protein-coding genetic variation in 60,706 humans. Nature *536*, 285–291.

Loh, P.-R., Danecek, P., Palamara, P.F., Fuchsberger, C., A Reshef, Y., K Finucane, H., Schoenherr, S., Forer, L., McCarthy, S., Abecasis, G.R., et al. (2016). Reference-based phasing using the Haplotype Reference Consortium panel. Nat. Genet. *48*, 1443–1448.

Maitra, A., Arking, D.E., Shivapurkar, N., Ikeda, M., Stastny, V., Kassauei, K., Sui, G., Cutler, D.J., Liu, Y., Brimble, S.N., et al. (2005). Genomic alterations in cultured human embryonic stem cells. Nat. Genet. *37*, 1099–1103.

Mallepaly, R., Butler, P.R., Herati, A.S., and Lamb, D.J. (2017). Genetic basis of male and female infertility. Monogr. Hum. Genet. *21*, 1–16.

Maples, B.K., Gravel, S., Kenny, E.E., and Bustamante, C.D. (2013). RFMix: A discriminative modeling approach for rapid and robust local-ancestry inference. Am. J. Hum. Genet. *93*, 278–288.

McKenna, A., Hanna, M., Banks, E., Sivachenko, A., Cibulskis, K., Kernytsky, A., Garimella, K., Altshuler, D., Gabriel, S., Daly, M., and DePristo, M.A. (2010). The genome analysis toolkit: a MapReduce framework for analyzing next-generation DNA sequencing data. Genome Res. *20*, 1297–1303.

McLaren, W., Gil, L., Hunt, S.E., Riat, H.S., Ritchie, G.R.S., Thormann, A., Flicek, P., and Cunningham, F. (2016). The Ensembl variant effect predictor. Genome Biol. *17*, 122.

Mei, Q., Li, X., Zhang, K., Wu, Z., Li, X., Meng, Y., Guo, M., Luo, G., Fu, X., and Han, W. (2017). Genetic and methylation-induced loss of miR-181a2/181b2 within chr9q33.3 facilitates tumor growth of cervical cancer through the PIK3R3/Akt/FoxO signaling pathway. Clin. Cancer Res. *23*, 575–586.

Merkle, F.T., and Eggan, K. (2013). Modeling human disease with pluripotent stem cells: From genome association to function. Cell Stem Cell *12*, 656–668.

Merkle, F.T., Ghosh, S., Kamitaki, N., Mitchell, J., Avior, Y., Mello, C., Kashin, S., Mekhoubad, S., Ilic, D., Charlton, M., et al. (2017). Human pluripotent stem cells recurrently acquire and expand dominant negative P53 mutations. Nature *545*, 229–233.

Merkle, F.T., Maroof, A., Wataya, T., Sasai, Y., Studer, L., Eggan, K., and Schier, A.F. (2015). Generation of neuropeptidergic hypothalamic neurons from human pluripotent stem cells. Development *142*, 633–643.

Mills, R.E., Walter, K., Stewart, C., Handsaker, R.E., Chen, K., Alkan, C., Abyzov, A., Yoon, S.C., Ye, K.K., Cheetham, R.K., et al. (2011). Mapping copy number variation by population-scale genome sequencing. Nature *470*, 59–65.

Mosher, J.T., Pemberton, T.J., Harter, K., Wang, C., Buzbas, E.O., Dvorak, P., Simón, C., Morrison, S.J., and Rosenberg, N.A. (2010). Lack of population diversity in commonly used human embryonic stem-cell lines. N. Engl. J. Med. *362*, 183–185.

Närvä, E., Autio, R., Rahkonen, N., Kong, L., Harrison, N., Kitsberg, D., Borghese, L., Itskovitz-Eldor, J., Rasool, O., Dvorak, P., et al. (2010). High-resolution DNA analysis of human embryonic stem cell lines reveals culture-induced copy number changes and loss of heterozygosity. Nat. Biotechnol. *28*, 371–377.

Nelson, P.T., Kondziolka, D., Wechsler, L., Goldstein, S., Gebel, J., DeCesare, S., Elder, E.M., Zhang, P.J., Jacobs, A., McGrogan, M., et al. (2002). Clonal human (hNT) neuron grafts for stroke therapy: neuropathology in a patient 27 months after implantation. Am. J. Pathol. *160*, 1201–1206.

Nguengang Wakap, S., Lambert, D.M., Olry, A., Rodwell, C., Gueydan, C., Lanneau, V., Murphy, D., Le Cam, Y., and Rath, A. (2020). Estimating cumulative point prevalence of rare diseases: analysis of the Orphanet database. Eur. J. Hum. Genet. *28*, 165–173.

Nguyen, H.T., Geens, M., Mertzanidou, A., Jacobs, K., Heirman, C., Breckpot, K., and Spits, C. (2014). Gain of 20q11.21 in human embryonic stem cells improves cell survival by increased expression of Bcl-xL. Mol. Hum. Reprod. *20*, 168–177.

Nicholls, R.D., Knoll, J.H.M., Butler, M.G., Karam, S., and Lalande, M. (1989). Genetic imprinting suggested by maternal heterodisomy in nondeletion Prader-Willi syndrome. Nature *342*, 281–285.

Nunes, J.M., Buhler, S., Roessli, D., Sanchez-Mazas, A., Andreani, M., Benhamamouch, S., Boldyreva, M., Canossi, A., Chiaroni, J., Darke, C., et al. (2014). The HLA-net GENE[RATE] pipeline for effective HLA data analysis and its application to 145 population samples from Europe and neighbouring areas. Tissue Antigens *83*, 307–323.

O'Flynn O'Brien, K.L., Varghese, A.C., and Agarwal, A. (2010). The genetic causes of male factor infertility: a review. Fertil. Steril. *93*, 1–12.

Pato, M.T., Sobell, J.L., Medeiros, H., Abbott, C., Sklar, B.M., Buckley, P.F., Bromet, E.J., Escamilla, M.A., Fanous, A.H., Lehrer, D.S., et al. (2013). The genomic psychiatry cohort: partners in discovery. Am. J. Med. Genet. *162*, 306–312.

Quang, D., Chen, Y., and Xie, X. (2015). DANN: a deep learning approach for annotating the pathogenicity of genetic variants. Bioinformatics *31*, 761–763.

Rehm, H.L., Berg, J.S., Brooks, L.D., Bustamante, C.D., Evans, J.P., Landrum, M.J., Ledbetter, D.H., Maglott, D.R., Martin, C.L., Nussbaum, R.L., et al. (2015). ClinGen – the clinical genome resource. N. Engl. J. Med. *372*, 2235–2242.

Ripke, S., Neale, B.M., Corvin, A., Walters, J.T.R., Farh, K.H., Holmans, P.A., Lee, P., Bulik-Sullivan, B., Collier, D.A., Huang, H., et al. (2014). Biological insights from 108 schizophrenia-associated genetic loci. Nature *511*, 421–427.

Robinson, J.T., Thorvaldsdóttir, H., Winckler, W., Guttman, M., Lander, E.S., Getz, G., and Mesirov, J.P. (2011). Integrative genomics viewer. Nat. Biotechnol. *29*, 24–26.

Rogers, M.F., Shihab, H.A., Mort, M., Cooper, D.N., Gaunt, T.R., and Campbell, C. (2018). FATHMM-XF: accurate prediction of pathogenic point mutations via extended features. Bioinformatics *34*, 511–513.

Rouhani, F., Kumasaka, N., de Brito, M.C., Bradley, A., Vallier, L., and Gaffney, D. (2014). Genetic background drives transcriptional variation in human induced pluripotent stem cells. PLoS Genet. *10*, e1004432.

Samson, M., Libert, F., Doranz, B.J., Rucker, J., Liesnard, C., Farber, C.M., Saragosti, S., Lapoumeroulie, C., Cognaux, J., Forceille, C., et al. (1996). Resistance to HIV-1 infection in caucasian individuals bearing mutant alleles of the CCR-5 chemokine receptor gene. Nature *382*, 722–725.

Sasaki, K., Yokobayashi, S., Nakamura, T., Okamoto, I., Yabuta, Y., Kurimoto, K., Ohta, H., Moritoki, Y., Iwatani, C., Tsuchiya, H., et al. (2015). Robust in vitro induction of human germ cell fate from pluripotent stem cells. Cell Stem Cell *17*, 178–194.

Shihab, H.A., Gough, J., Cooper, D.N., Day, I.N.M., and Gaunt, T.R. (2013). Predicting the functional consequences of cancer-associated amino acid substitutions. Bioinformatics *29*, 1504–1510.

Sittig, L.J., Carbonetto, P., Engel, K.A., Krauss, K.S., Barrios-Camacho, C.M., and Palmer, A.A. (2016). Genetic background limits generalizability of genotype-phenotype relationships. Neuron *91*, 1253–1259.

Solomon, B.D., Nguyen, A.-D., Bear, K.A., and Wolfsberg, T.G. (2013). Clinical genomic database. Proc. Natl. Acad. Sci. USA *110*, 9851–9855.

Sudmant, P.H., Rausch, T., Gardner, E.J., Handsaker, R.E., Abyzov, A., Huddleston, J., Zhang, Y., Ye, K., Jun, G., Fritz, M.H.Y., et al. (2015). An integrated map of structural variation in 2,504 human genomes. Nature *526*, 75–81.

Tate, J.G., Bamford, S., Jubb, H.C., Sondka, Z., Beare, D.M., Bindal, N., Boutselakis, H., Cole, C.G., Creatore, C., Dawson, E., et al. (2019). COSMIC: the catalogue of somatic mutations in cancer. Nucleic Acids Res. *47*, D941–D947.

Trounson, A., and DeWitt, N.D. (2016). Pluripotent stem cells progressing to the clinic. Nat. Rev. Mol. Cell Biol. *17*, 194–200.

Venkatesh, T., Suresh, P.S., and Tsutsumi, R. (2014). New insights into the genetic basis of infertility. Appl. Clin. Genet. *7*, 235–243.

Wang, L., Meece, K., Williams, D.J., Lo, K.A., Zimmer, M., Heinrich, G., Martin Carli, J., Leduc, C.A., Sun, L., Zeltser, L.M., et al. (2015). Differentiation of hypothalamic-like neurons from human pluripotent stem cells. J. Clin. Invest. *125*, 796–808.

Yamamoto, F.I., Clausen, H., White, T., Marken, J., and Hakomori, S.I. (1990). Molecular genetic basis of the histo-blood group ABO system. Nature *345*, 229–233.

Yang, J., Lee, S.H., Goddard, M.E., and Visscher, P.M. (2011). GCTA: a tool for genome-wide complex trait analysis. Am. J. Hum. Genet. *88*, 76–82.

Yilmaz, A., Peretz, M., Aharony, A., Sagi, I., and Benvenisty, N. (2018). Defining essential genes for human pluripotent stem cells by CRISPR-Cas9 screening in haploid cells. Nat. Cell Biol. *20*, 610–619.

Yizhak, K., Aguet, F., Kim, J., Hess, J.M., Kübler, K., Grimsby, J., Frazer, R., Zhang, H., Haradhvala, N.J., Rosebrock, D., et al. (2019). RNA sequence analysis reveals macroscopic somatic clonal expansion across normal tissues. Science *364*, eaaw0726.

Zhao, M., Kim, P., Mitra, R., Zhao, J., and Zhao, Z. (2016). TSGene 2.0: an updated literature-based knowledgebase for Tumor Suppressor Genes. Nucleic Acids Res. *44*, D1023–D1031.

Zhou, Q.Q., Wang, M., Yuan, Y., Wang, X., Fu, R., Wan, H., Xie, M., Liu, M., Guo, X., Zheng, Y., et al. (2016). Complete meiosis from embryonic stem cell-derived germ cells in vitro. Cell Stem Cell *18*, 330–340.

STAR★METHODS

KEY RESOURCES TABLE

REAGENT or RESOURCE	SOURCE	IDENTIFIER
Critical commercial assays		
Nextera DNA Flex Library Prep Kit (96 samples) for whole genome sequence library preparation by tagmentation	Illumina, Inc	Cat# 20018705
Nextera DNA CD Indexes (96 indexes, 96 samples)	Illumina, Inc	Cat# 20018708
Deposited data		
Whole genome sequencing data of all cell lines included in the study, except those from UCLA which cannot be shared due to legal restrictions	This paper	https://duos.broadinstitute.org/, dataset DUOS-000121
Experimental models: Cell lines		
Details of human embryonic stem cell lines from multiple institutions used in this study are provided in Table S1	This paper	This paper
Software and algorithms		
Picard	Open-source, developed at the Broad Institute of MIT and Harvard	https://broadinstitute.github.io/picard/
GATK	McKenna et al. (2010)	GATK nightly-2015-07-31-g3c929b0
PLINK 2.0	Chang et al. (2015)	https://www.cog-genomics.org/plink2
GCTA	Yang et al. (2011)	https://yanglab.westlake.edu.cn/software/gcta
Genome STRiP version 2.0, r2.00.1587	Handsaker et al. (2015)	http://www.broadinstitute.org/software/genomestrip/
Eagle v2.4.1	Loh et al. (2016)	https://alkesgroup.broadinstitute.org/Eagle/
MoChA v2020-09-01	Open-source, developed at the Broad Institute of MIT and Harvard by Giulio Genovese under the supervision of Steven McCarroll.	https://github.com/freeseek/mocha https://github.com/freeseek/mocha
VEP version 85	McLaren et al. (2016)	https://www.ensembl.org/info/docs/tools/vep/index.html
Hail	Open-source, developed at the Broad Institute of MIT and Harvard	https://hail.is
LOFTEE (plugin to VEP)	Karczewski et al. (2020)	https://github.com/konradjk/loftee
ClinVar	Landrum et al. (2014)	www.ncbi.nlm.nih.gov/clinvar/
CADD	Kircher et al. (2014)	https://cadd.gs.washington.edu/
DANN	Quang et al. (2015)	https://cbcl.ics.uci.edu/public_data/DANN/
COSMIC	Tate et al. (2019)	https://cancer.sanger.ac.uk/cosmic/
HSCGP data portal implementation code and documentation	This paper	https://github.com/broadinstitute/hscgp (https://doi.org/10.5281/zenodo.5794210)
FATHMM-XF	Rogers et al. (2018)	https://fathmm.biocompute.org.uk/
FATHMM cancer	Shihab et al. (2013)	https://fathmm.biocompute.org.uk/
OMIM database of genes and variants associated with Mendelian disorders	Online Mendelian Inheritance in Man, OMIM®. McKusick-Nathans Institute of Genetic Medicine, Johns Hopkins University (Baltimore, MD), 2019	http://www.omim.org

(*Continued on next page*)

Continued

REAGENT or RESOURCE	SOURCE	IDENTIFIER
ClinGen curated database of genes and variants associated with human disease	Rehm et al. (2015)	http://www.ncbi.nlm.nih.gov/projects/dbvar/clingen/
Curated dosage-sensitive genes from the American College of Medical Genetics (ACMG)	Rehm et al. (2015)	https://www.ncbi.nlm.nih.gov/projects/dbvar/clingen/acmg.shtml
Genimprint database of imprinted genes		http://www.geneimprint.com/s
TSgene database of tumor suppressor genes	Zhao et al. (2016)	https://bioinfo.uth.edu/TSGene/
KEGG p53 pathway	Kanehisa and Goto (2000)	https://www.genome.jp/dbget-bin/www_bget?pathway+hsa04115
Clinical Genomic Database (CGD)	Solomon et al. (2013)	https://research.nhgri.nih.gov/CGD/
ExAC	Lek et al. (2016)	https://gnomad.broadinstitute.org/
gnomAD v2.1	Karczewski et al. (2020)	https://gnomad.broadinstitute.org/
Other		
Human stem cell genome project (HSCGP) data portal developed to allow users to browse whole genome sequencing data from hESC lines included in this study	This paper	https://hscgp.broadinstitute.org/hscgp

RESOURCE AVAILABILITY

Lead contact

Further information and requests for resources should be directed to and will be fulfilled by lead contact, Florian T. Merkle (fm436@medschl.cam.ac.uk).

Materials availability

This study did not generate new unique reagents.

Data and code availability

Whole genome sequencing data (in CRAM format) from cell lines included in this study have been deposited at https://duos.broadinstitute.org/, and are available as of the date of publication if access is granted. The accession number (dataset DUOS-000121) is also listed in the key resources table. Access to this dataset is managed to ensure the preservation of donor anonymity, and data from cell lines obtained from the University of California, Los Angeles cannot be shared by us due to legal restrictions. To request access, follow the instructions of the DUOS website or in case of difficulties contact Anna Neumann (neumann@broadinstitute.org). All original code is publicly available as of the date of publication (10.5281/zenodo.5794210, please see also key resources table).

EXPERIMENTAL MODEL AND SUBJECT DETAILS

Human embryonic stem cell lines included in this study were all derived under informed consent as confirmed by Harvard University at the time of import, and either deposited on the NIH registry of embryonic stem cell lines or registered with the UK Stem Cell Steering Committee. Details of cell lines in this study, including their Research Resource Identifiers (RRIDs), are listed in Table S1. We took care to authenticate cell lines wherever data were available from providing institutions, and noticed that samples MShef5 and MShef7 were swapped (this has been corrected in our files), and that the XX genotypes of WIBR1 and WIBR6 in this study did not match their reported XY genotype. We were unable to ascertain the source of this discrepancy due to the lack of ground truth data and have included a flag in TableS1 to caution users accordingly.

Cell lines that had not been prepared for potential clinical use were acquired and cultured as previously described (Merkle et al., 2017). Briefly, all cultures were tested for the presence of mycoplasma and cultured in a humidified 37°C tissue culture incubator in the presence of 5% CO_2 and 20% O_2. Cell lines were adapted to a common set of culture media by being thawed in the presence of 10 μM Rock inhibitor (Y-27632 DNSK International) into either a 1:1 mixture of DMEM-based medium supplemented with knockout serum replacement (KSR), and mTeSR1 (Stemcell Technologies) on a substrate of Matrigel (Corning), or KOSR medium on a irradiated mouse embryonic fibroblasts (MEFs). Cultures were fed daily in the absence of antibiotics or Rock inhibitor, and once homogeneous cultures with pluripotent stem cell morphology and lacking spontaneous differentiation had been established, they were maintained in KSR–mTeSR1, passaged with 0.5 mM EDTA in calcium- and magnesium-free PBS followed by gentle trituration

in KSR–mTeSR1 medium containing 10 μ M Y-27632 and re-plating onto Matrigel-coated plates. Cell lines were frozen down in KSR–mTeSR1 medium containing a final concentration of 10% DMSO and 40% sterile heat-inactivated fetal bovine serum.

METHOD DETAILS

Whole genome sequencing and genotyping

Cell pellets of approximately 1-5 million cells were digested overnight at 50°C in 500 μl lysis buffer containing 100 μg/ml proteinase K (Roche), 10 mM Tris pH 8.0, 200 mM NaCl, 5% w/v SDS, 10 mM EDTA, followed by Phenol:Chloroform precipitation, ethanol washes, and resuspension in 10 mM Tris buffer (pH 8.0). Genomic DNA from hESCs was processed into "tagmented" Illumina Nextera Flex libraries. Control whole genomes used to set the FDR for CNV calling were sequenced from libraries prepared by shearing 100 ng input genomic DNA in 50 μL of solution. For adapter ligation, Illumina paired end adapters were replaced with palindromic forked adapters with unique 8 base index sequences embedded within the adapter. Size selection was performed using Sage's Pippin Prep, with a target insert size of 370bp +/- 10%. Both hESC and control samples were sequenced at the Genomics Platform at the Broad Institute of MIT and Harvard. All sample information tracking was performed by automated LIMS messaging. Libraries were quantified using quantitative PCR (KAPA biosystems) with probes specific to the ends of the adapters. This assay was automated using Agilent's Bravo liquid handling platform. Based on qPCR quantification, libraries were normalized to 1 nM. Samples were then combined with HiSeq X Cluster Amp Mix 1, 2, and 3 into single wells on a strip tube using the Hamilton Starlet Liquid Handling system. Cluster amplification of the templates was performed according to the manufacturer's protocol (Illumina) using the Illumina cBot. Flowcells were sequenced on HiSeqX, then analyzed using RTA2. The target sequencing depth had a median >25x coverage with paired-end 151 base reads. Sequence data were processed using the Picard pipeline to yield BAM files aligned to the hg19 reference genome using best practices from GATK software (McKenna et al., 2010). Data from each cell line was independently processed with the HaplotypeCaller walker and further aggregated with the CombineGVCFs and GenotypeGVCFs walkers. Genotyped sites were filtered using the ApplyRecalibration walker.

Ancestry, relatedness, and HLA analysis

Genotypes from the cell lines at sites in common with sites genotyped in the 1000 Genomes Project Phase 1 (1000 Genomes Project Consortium, 2012) and with a minor allele frequency (MA) of at least 1% were selected for relatedness and ancestry analysis. For relatedness analysis, selected sites were pruned using PLINK 2.0 software (Chang et al., 2015, "–indep 50 5 2" command option) and estimates for the amount of IBD1 and IBD2 regions were computed ("–genome gz" command option). Sample pairs were considered directly related (parent-child or full sibling) when estimates were between 35% and 70%. For the sibling pairs we identified, IBS0 values ranged from 0.0019 to 0.0061 and Kinship values ranged from 0.2058 to 0.2845 (Table S1B) with the exception of the likely half-sibling lines UCLA8 and UCLA9 (Figure 2C).

For ancestry analysis, selected sites were extracted from the dataset, merged with 1000 Genomes Project genotypes, and pruned using PLINK 2.0 software ("–indep 50 5 2" command option). Principal component analysis was then performed for this combined dataset by computing the pairwise relationship matrix across all subjects (using the plink command "–make-grm-bin"), and computing the principal components using GCTA software (Yang et al., 2011). Global ancestral components for European, African, Native American, and Asian ancestry were estimated from the first three principal components using a linear model trained by assigning full European, African, and Asian ancestry to the appropriate 1000 Genomes population samples, and assigning estimated ancestry proportions to Latino samples using available published estimates (1000 Genomes Project Consortium, 2012; Maples et al., 2013). Human Leukocyte Antigen (HLA) genotype was ascertained by genotyping hESCs for SNPs associated with HLA haplotypes in the CEU ethnic group (De Bakker et al., 2006).

Polygenic risk score (PRS) computations

To compute PRSs, we used summary statistics from studies performed on individuals predominantly of European ancestry. For each phenotype with available summary statistics, we computed the score for all available markers with association p-values in the original study (Bulik-Sullivan et al., 2015) that were less than 0.5 (e.g. 1,218,732 for BMI). PRS's were computed with established methodologies (Purcell et al., 2009) and PLINK 2.0 software (Chang et al., 2015) using genotypes from WGS data. To control for potential methodological biases, we also calculated PRSs using high-coverage WGS data from unrelated human samples including 383 schizophrenia (SCZ) cases and 489 SCZ controls (Ripke et al., 2014) and adjusted raw PRSs by regressing the first 12 principal component values to correct for slight biases introduced in the computation of the summary statistics. Principal components beyond the first 12 did not show significant correlation with raw PRS. Raw PRS values were normalized such that the distribution of PRS scores from SCZ control subjects had zero mean and unitary standard deviation (SD).

CNV calling from read depth variation

Copy number variants were ascertained and genotyped using Genome STRiP version 2.0 (r2.00.1587, http://www.broadinstitute.org/software/genomestrip/), using the CNVDiscoveryPipeline with default settings (Handsaker et al., 2015). This software examines average depth of coverage (DOC) genome-wide to identify chromosomes and genomic regions whose normalized DOC deviated from the expected copy number of 2 for autosomes, and 2 or 1 for the X and Y chromosomes. After initial CNV calling, quality was assessed using signal intensity data from Omni 2.5 Microarrays run on 500 individuals from a control cohort (Pato et al.,

2013). Using the IRS method (Handsaker et al., 2015) we established separate length thresholds for deletions (length threshold = 1117) and duplications (length threshold = 2750) that achieved a false discovery rate under 3% for both categories of CNV in these control data (Figure S3A). By comparing the number of CNVs called in the cell lines to CNVs called in the control cohort, stratified by CNV type, length and ancestry, we reasoned that the false discovery rate in the cell lines should be comparable to the estimated false discovery rate of 3% in the control cohort. To test this assumption, we sequenced 121 hESC lines using the Infinium PsychArray (> 500,000 probes), filtered out probes with high intrinsic variability in intensity, and asked which small CNV calls made by DOC were supported by probe intensity data from CNVs that contained >1 or >2 DNA microarray probes.

To increase CNV calling power and to enhance quality control, CNV analysis was performed on a combined cohort of 130 sequenced hESC lines and a control cohort consisting of 234 human samples from primary blood that had undergone WGS on the same platform (Illumina HiSeqX) and to similar depth as the hESC lines. These control samples were selected from the larger cohort of 500 samples based on their absence of a psychiatric diagnosis (i.e. controls) and their origin from blood as opposed to lymphoblastoid cell lines to avoid any potential confounds from disease- or somatic mutation-associated CNVs (Pato et al., 2013). We then separated CNVs into small (1117 bp - 1 Mbp) and large (> 1 Mbp) categories since large variants were uncommon among similarly-sequenced human samples and therefore more likely to be culture-acquired and potentially deleterious, and manually confirmed all large CNV calls from hESCs and human control samples.

Identification of large structural variants

To detect large-scale copy number alterations (> 1 Mbp) by sequencing depth of coverage (DOC), we scanned the genome for segments where one sample was enriched or depleted in depth of sequencing coverage compared to the other cell line samples. We divided the genome into non-overlapping 100 kbp bins of uniquely-alignable sequence (based on 101bp k-mers) and computed the normalized depth of coverage using Genome STRiP (Handsaker et al., 2015). For each contiguous range of bins, a Z-score was computed for each sample over the genomic interval as:

$$Z(S,I) = \mathrm{abs}(\mathrm{coverage}(S,I) - \mathrm{median}(I) / \mathrm{MAD}(I))$$

where coverage(S,I) is the normalized depth of coverage for sample S over the interval, median(I) is the median coverage over all samples and MAD is the median-absolute-deviation. We performed a heuristic search to identify candidate CNV intervals with a Z-score > 3 that were local maxima for sample S. We required that candidate CNV intervals have sharp boundaries, such that the depth of coverage for sample S in the next adjacent bin (on both ends of the interval) was more than halfway from the coverage for S inside the interval to the median coverage of the adjacent bin outside the interval. The final set of large CNVs reported were selected to span at least 10 bins (minimum length 1 Mbp) and have a Z-score > 5. The boundaries of each CNV were manually reviewed and adjusted as needed near centromeres or telomeres or to merge adjacent calls. Contiguous but compound variants were considered as one unique event.

To determine if CNVs detected in hESCs were present in all sequenced cells or in a subset of them, we calculated the likelihood that the divergence from an integer copy number could have arisen by chance. We set the P value threshold for detecting mosaicism at 1×10^{-3} since human blood samples had only 2/243 (< 1%) samples with smaller P values (Figure S3B), and otherwise classified CNVs as fixed. CNV calls made from sequencing depth of coverage analysis were supported by allelic coverage at heterozygous sites. This was measured at all heterozygous SNPs observed at least five times across the whole dataset and falling outside of regions exhibiting excessive heterozygosity in the 1000 Genomes project dataset, as these regions might be more prone to mismapping. Each remaining heterozygous allele was then phased using Eagle v2.4.1 +htslib-1.9 (Loh et al., 2016). Phased genotypes and allelic coverages were then analyzed for allelic imbalances with MoChA version 2020-09-01. Highly confident calls (LOD>20) of large structural variations (> 1 Mbp) were reported (Table S3). To distinguish acquired CN-LOH events from fixed runs of homozygosity arising from inbreeding or complex chromosomal rearrangement, we considered only events that were mosaic and extended to the telomere, consistent with CN-LOH formation in culture by a single mitotic crossover event.

Validation of large structural variants

All structural variant calls made by SNP DNA microarray in a published dataset (Canham et al., 2015) were compared to those 500 kbp or larger that were called in this study by DOC analysis. Calls made by SNP DNA microarray had been mapped to the hg38 reference genome, so variant calls were converted to hg19 coordinates to enable comparison to WGS data. We considered the 20 variants detected by Canham and colleagues regardless of their size, and found that 15 of them were confirmed by WGS data. Manual inspection of the five calls made only by SNP DNA microarray showed that three of these calls were likely not true CNVs, and two were likely explained by the divergent culture histories of cell lines that were sequenced at different passages.

Detection and interpretation of small CNVs

Regions containing large fixed or mosaic CNVs were excluded from small-scale CNV analysis in affected samples to reduce the potential "fragmentation" of large CNVs into smaller calls. Singleton events were defined as being present only once in any of the other hESC lines, human samples, or in previously described databases such as the 1000 Genomes Project (1000 Genomes Project Consortium, 2012).

To ask whether hESCs might be enriched for specific smaller CNVs, we examined the genome-wide distribution of CNVs < 1 Mbp in hESCs and removed all but one cell line from the sib-ships we identified to control for CNVs shared between first-degree relatives.

We measured the frequency of each CNV found in the hESCs in two control cohorts: 212 human samples of European ancestry (GPC1) and 250 human samples of Latino or African American ancestry (CPG2). We identified events that were present in at least 5 hESC lines but that were absent from the European control samples and found in less than 10% of the other control samples. This analysis identified the known region on Chr20q11.21 as well as four other potentially differentiated CNVs. Manual review of these four additional regions revealed that they are all in small regions of extreme GC-content with excessive sequencing coverage and not likely to be real CNVs.

For each cell line, genes overlapping with 5997 CNVs were identified and annotated using the GeneOverlap module in Genome-Strip 2.0 and GENCODE. The region of overlap (coding vs. exonic vs. intronic) and the type of CNV (deletion or duplication) were also recorded. 2185 CNVs contained at least some part of an annotated gene (Table S3). Limiting CNV overlaps to coding regions and UTRs reduced these numbers to 357 deleted (CN < 2) and 321 duplicated (CN > 2) genes, respectively.

Identification of SNVs in hESCs

To ensure reliable assignment of variant function and to confidently identify potentially high impact variants, genotypes were called jointly in all hESC lines and genotype data were annotated using Variant Effect Predictor version 85 (VEP, McLaren et al., 2016) in Hail (https://hail.is) with the Loss-of-function Transcript Effect Estimator plugin (LOFTEE, Karczewski et al., 2020). Candidate variants called by VEP were advanced for further analysis and false positive calls were removed from the VEP variant call set by applying similar filters used by gnomAD to identify ‘‘high confidence and high quality’’ (HC-HQ) variant calls (Karczewski et al., 2020). Specifically, we split multiallelic sites and discarded all calls without a ‘‘PASS’’ filter tag as determined by the Variant Quality Score Recalibration tool (GATK). We also excluded variant sites where not a single sample had high genotyping quality as defined by (Depth of Coverage >= 10, Genotype Quality >= 20 and Minor allele fraction >= 0.2). Variants in low complexity regions and segmental duplications were filtered out and only variants that met the gnomAD ‘‘PASS’’ filter criteria or were missing were retained. These filtering steps resulted in a total of 15.5M high quality variants corresponding to approximately 70% of all variants in the data.

Since VEP annotates individual transcripts, only variants on canonical transcripts as defined by Gencode/Ensembl were included in downstream analyses. Variants were binned into synonymous, missense and lofs using the following criteria:

LOFS: "frameshift_variant", "splice_acceptor_variant", "splice_donor_variant", "stop_gained"

Missense: "inframe_deletion", "inframe_insertion", "missense_variant", "start_lost", "stop_lost", "protein_altering_variant"

Synonymous: "synonymous_variant", "stop_retained_variant", "incomplete_terminal_codon_variant"

To exclude common variants and to limit our analyses to biologically variant categories, the remaining calls were restricted to missense and LOF variants with an allele frequency less than 0.001 in ExAC that were bioinformatically predicted to be deleterious by both CADD (CADD-phred >20) and DANN (>0.99). ClinVar (Landrum et al., 2014) and COSMIC coding mutations (Tate et al., 2019) were used to further refine the call set to disease relevant variants. Variant interpretation and prioritization was performed using a series of variant-level and gene level filters described below (‘SNV characterization and prioritization’).

Data portal architecture and implementation

The interactive data portal will run on most operating systems, and was implemented using software freely available at https://github.com/broadinstitute/hscgp. We hope this platform will lay a foundation for interested users to readily produce similar portals to host their own data. The portal is implemented using Ruby on Rails and uses mongodb for object storage. Docker images are provided for the Ruby on Rails application and for the mongodb database and these can both be run on any machine or cluster that supports running docker containers and networking between thems. We utilize the firecloud API to allow sensitive genomic data to be accessed securely from its storage location on a protected Google Cloud bucket. This architecture ensures that authenticated users of the application can access loci of interest across genomes of different cell lines without exposing a complete BAM file or passing unencrypted genomic data between machines.

SNV characterization and prioritization

We used a series of gene-level and variant-level filters to identify SNVs of particular interest to human disease, as described in the main manuscript text. These filters are derived from publicly-available databases and bioinformatic prediction algorithms and represented in the columns of Table S4 and detailed below:

Variant details

gene, HUGO gene nomenclature committee (HGNC) official human gene symbol (https://www.genenames.org/);

transcript, Ensembl transcript identifier (https://www.ensembl.org/);

locus, genomic coordinates from hg19 reference, and base of the reference and alternate allele;

chr, human chromosome on which the variant is located;

pos, chromosomal position of the variant in hg19 coordinates;

ref, reference base at this chromosomal position;

alt, alternate base at this chromosomal position;

duplicated_locus, TRUE if the site is multiallelic and has been split.

rsid, reference SNP identifier from dbSNP (https://www.ncbi.nlm.nih.gov/snp/);

worst_csq, worst consequence predicted by VEP

consequence, consequence predicted by VEP

hgvsp, Ensembl identifier of the affected protein and most likely amino acid change;
hgvsc, Ensembl identifier of the affected transcript and coding change;
homs, file name(s) of hESC line(s) in which the variant was identified in a homozygous state;
hom_count, number of homozygous variant among the 143 analyzed hESC lines;
hets, file name(s) of hESC line(s) in which the variant was identified in a heterozygous state;
het_count, number of heterozygous variant among the 143 analyzed hESC lines;
AC, total number of variant alleles genotyped among the 143 analyzed hESC lines;
AN, total number of alleles at that genomic location genotyped among the 143 analyzed hESC lines;
AF, ratio of variant to total alleles in hESCs;

Variant-level filters

gnomad_global_AC, number of times this variant was present in 15,708 whole genome sequences currently represented in the gnomAD database (Karczewski et al., 2020, https://gnomad.broadinstitute.org/);

gnomad_global_AF, ratio of variant to total alleles among the 15,708 whole genome sequences in gnomAD;

exome_global_AC, number of times this variant was present in the ExAC database in 60,706 exomes that are non-overlapping with the whole genomes represented in gnomAD (Lek et al., 2016, https://gnomad.broadinstitute.org/)

exome_global_AF, ratio of variant to total alleles at this genomic location in ExAC;

cadd_raw, raw score from the Combined Annotation Dependent Depletion (CADD) predictor of variant deleteriousness (Kircher et al., 2014, https://cadd.gs.washington.edu/);

cadd_phred, scaled phred-like CADD score where the bottom 90% of deleterious variants have a score of 0-10, the next 9% have scores of 10-20, and so on;

dann, score of variant deleteriousness from the Deleterious Annotation of genetic variants using Neural Networks (DANN) with scores ranging from 0 to 1 for neutral to most deleterious (Quang et al., 2015, https://cbcl.ics.uci.edu/public_data/DANN/);

fathmm_xf_cod_score, score of likely coding variant pathogenicity from the Functional Analysis Through Hidden Markov Models (FATHMM-XF) predictor (Rogers et al., 2018, https://fathmm.biocompute.org.uk/);

fathmm_xf_nc_score, FATHMM prediction for non-coding (e.g. splice donor) variants;

fathmm_warn, variant annotation from FATHMM at default sensitivity and specificity thresholds (https://fathmm.biocompute.org.uk/);

fathmm_cancer, FATHMM prediction of cancer-associated coding variants with annotations at default sensitivity and specificity thresholds (Shihab et al., 2013, http://fathmm.biocompute.org.uk/cancer.html);

cosmic_id, Catalogue Of Somatic Mutations in Cancer (COSMIC) numerical internal database identifier (Tate et al., 2019, http://cancer.sanger.ac.uk/);

cosmic_count, number of times the specific variant was reported in COSMIC, where multiple entries denote multiple splice isoforms;

cosmic_cds, base changes for each of the major splice isoforms of the protein in COSMIC,

cosmic_aa, amino acid changes for each of the major splice isoforms of the protein in COSMIC,

Gene-level filters

clin_def, clinical syndrome(s) associated with defects in the affected gene as reported in the manually-curated ClinVar database (Landrum et al., 2014, www.ncbi.nlm.nih.gov/clinvar/);

clin_sig, likely clinical significance of the variant based on manual curation of the strength of supporting data in the literature;

clin_vc, variant type;

clin_db, links to databases describing clinical syndrome(s) in greater detail;

MIM, identifier for the clinical syndrome(s) associated with defects the affected gene as reported in the manually-curated Online Mendelian Inheritance in Man database (http://www.omim.org/downloads, 2019);

Genomic.Location, genomic location of the gene associated with the clinical syndrome(s) described in OMIM;

Haploinsufficiency.Description, annotation of the gene as dosage-sensitive in the manually-curated ClinGen database (Rehm et al., 2015, http://www.ncbi.nlm.nih.gov/projects/dbvar/clingen/);

Loss.phenotype.OMIM.ID, OMIM entries for genes in which gene loss of function is associated with a clinical phenotype;

Dosage, annotation of haploinsufficiency among the 59 genes designated by the American College of Medical Genetics (ACMG) manual (https://www.ncbi.nlm.nih.gov/projects/dbvar/clingen/acmg.shtml),

Fertility_Related, genes described in the literature to be related to fertility (Mallepaly et al., 2017; O'Flynn O'Brien et al., 2010; Venkatesh et al., 2014);

Imprinted, inclusion of the gene in a manually-curated list of imprinted human genes (http://www.geneimprint.com/site/genes-by-species);

X-linked dominant, OMIM disease genes that show X-linked dominant inheritance;

Tumor Suppressors, inclusion of the gene in a curated list of tumor suppressors (Zhao et al., 2016, https://bioinfo.uth.edu/TSGene/);

COSMIC_tier, inclusion of the gene as a COSMIC Tier 1 gene that has an established association to cancer (Tate et al., 2019, http://cancer.sanger.ac.uk/census/);

P53_Pathway, inclusion of the gene in the KEGG p53 pathway (Kanehisa and Goto, 2000, https://www.genome.jp/dbget-bin/www_bget?pathway+hsa04115);

Growth restricting, Top 50 growth restricting genes in hESCs identified by (Yilmaz et al., 2018);

CONDITION, clinical syndrome associated with the gene in the Clinical Genomic Database (CGD: Solomon et al., 2013, https://research.nhgri.nih.gov/CGD/);

INHERITANCE, reported inheritance of the clinical syndrome associated with the gene in CGD;

COMMENTS, comments associated with the clinical syndrome associated with the gene in CGD;

INTERVENTION.RATIONALE, clinical intervention rationale for the clinical syndrome associated with the gene in CGD;

REFERENCES, references associated with the clinical syndrome associated with the gene in CGD;

Autosomal Dominant, OMIM disease genes that show dominant inheritance;

obs_mis, observed number of missense variants in this gene in gnomAD;

exp_mis, expected number of missense variants in this gene in gnomAD;

mis_z, ExAC score of gene constraint to missense variants where positive scores indicated increased constraint;

oe_mis_lower, 90% confidence interval for the lower bound of observed to expected missense variants in this gene in gnomAD;

oe_mis_upper, 90% confidence interval for the upper bound of observed to expected missense variants in this gene in gnomAD;

obs_lof, observed number of loss-of-function variants in a gene in gnomAD;

exp_lof, expected number of loss-of-function variants in a gene in gnomAD;

pLI, probability of loss intolerance from a LoF mutation from the ExAC database based on expected versus observed LoF mutations;

oe_lof, mean fraction of observed to expected loss-of-function variants in a given gene;

oe_lof_lower, 90% confidence interval for the lower bound of observed to expected loss-of-function variants in this gene in gnomAD;

oe_lof_upper, 90% confidence interval for the upper bound of observed to expected loss-of-function variants (LOEUF) in this gene in gnomAD;

constraint_flag, Flags assigned to constrained genes as defined in gnomad v2.1 (see https://gnomad.broadinstitute.org/faq)

gene_type, Type of constrained gene as per gnomad v2.1 (see https://gnomad.broadinstitute.org/faq)

QUANTIFICATION AND STATISTICAL ANALYSIS

Statistical analysis used is described in the main text or Methods Details above.

Cell Stem Cell, Volume *29*

Supplemental Information

Whole-genome analysis of human embryonic stem cells enables rational line selection based on genetic variation

Florian T. Merkle, Sulagna Ghosh, Giulio Genovese, Robert E. Handsaker, Seva Kashin, Daniel Meyer, Konrad J. Karczewski, Colm O'Dushlaine, Carlos Pato, Michele Pato, Daniel G. MacArthur, Steven A. McCarroll, and Kevin Eggan

Supplementary figures and legends

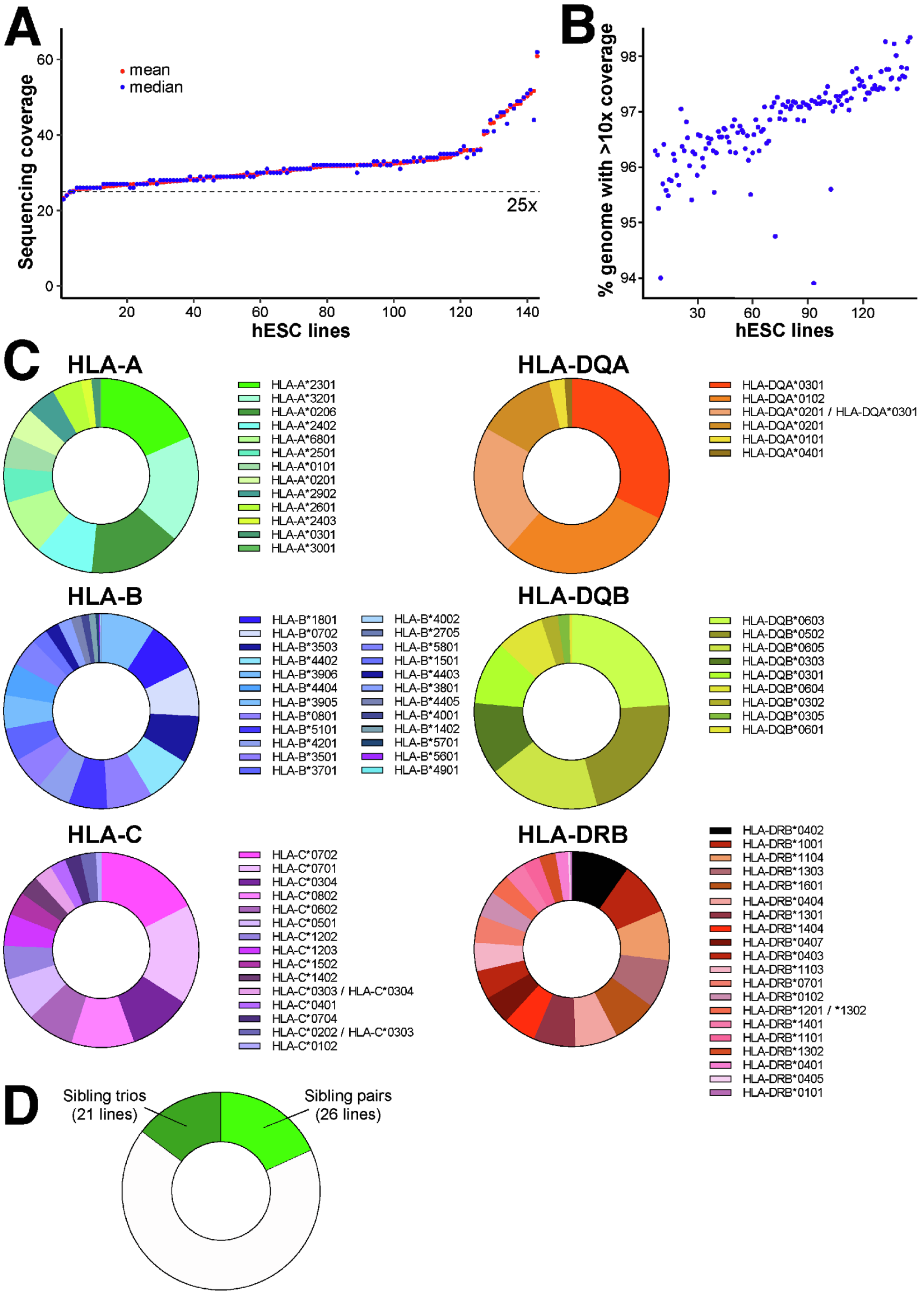

Figure S1: Sample sequencing coverage and diversity, related to main Figures 1 and 2. A) Whole genome sequencing coverage exceeded a mean and median of 25x for 140/143 human embryonic stem cell (hESC) lines, sorted by mean sequencing coverage. **B)** Percentage of the aligned genome sequenced to at least 10x mean coverage for 143 hESC lines sorted as in A. **C)** The human leukocyte antigen (HLA) haplotype diversity of 121 analyzed lines is consistent with their predominantly European origin. **D)** Approximately one third of sequenced hESC lines share sibling relationships.

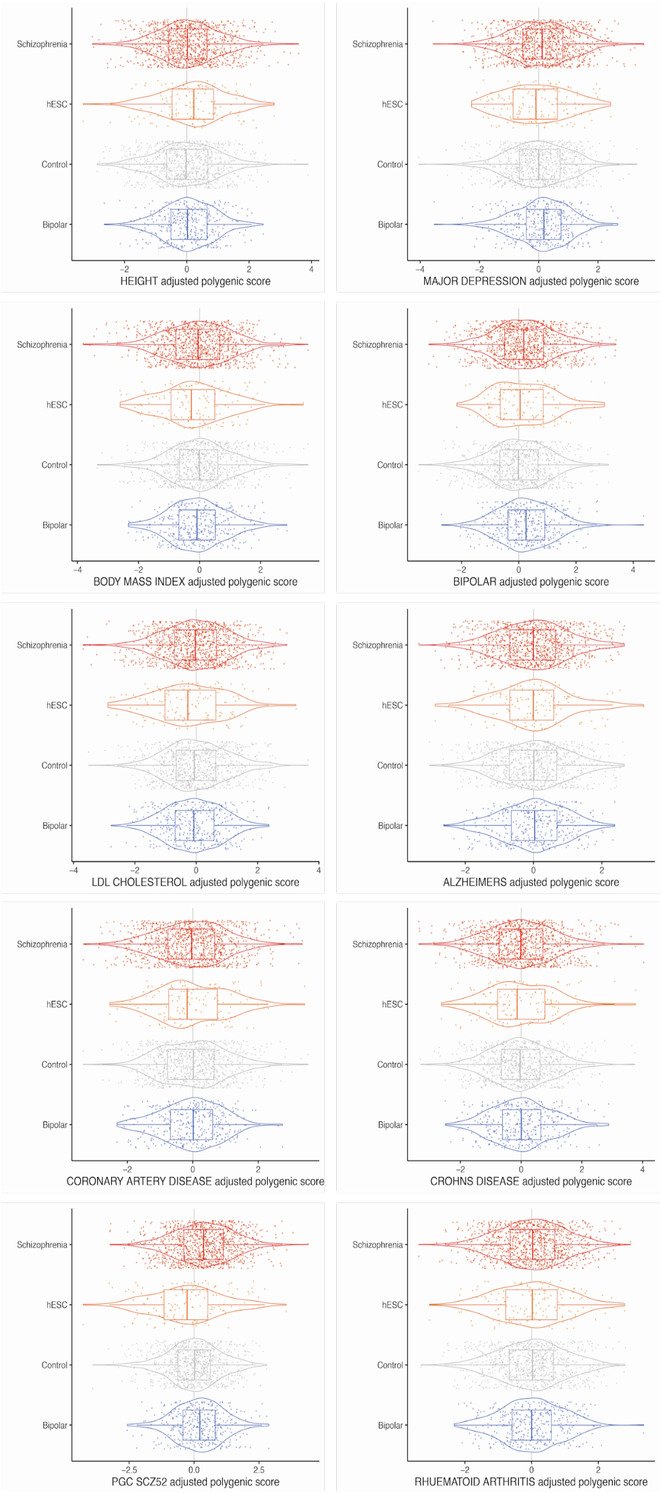

Figure S2: PRS distributions for hESCs and control samples, related to main Figure 3. Polygenic risk scores (PRS) were computed for hESCs and control samples from individuals with no diagnosed mental disorder or with either schizophrenia or bipolar disorder, normalized so that the control distribution had a mean of 0 and standard deviation (SD) of 1, and compared to reveal hESC lines that deviated more than 2 SD from the mean. Note that the only distribution that significantly deviates from the mean is the schizophrenia cohort when PRSs for schizophrenia were calculated (lower left). LDL, low density lipoprotein; PGC SCZ52, schizophrenia from the psychiatric genomics consortium.

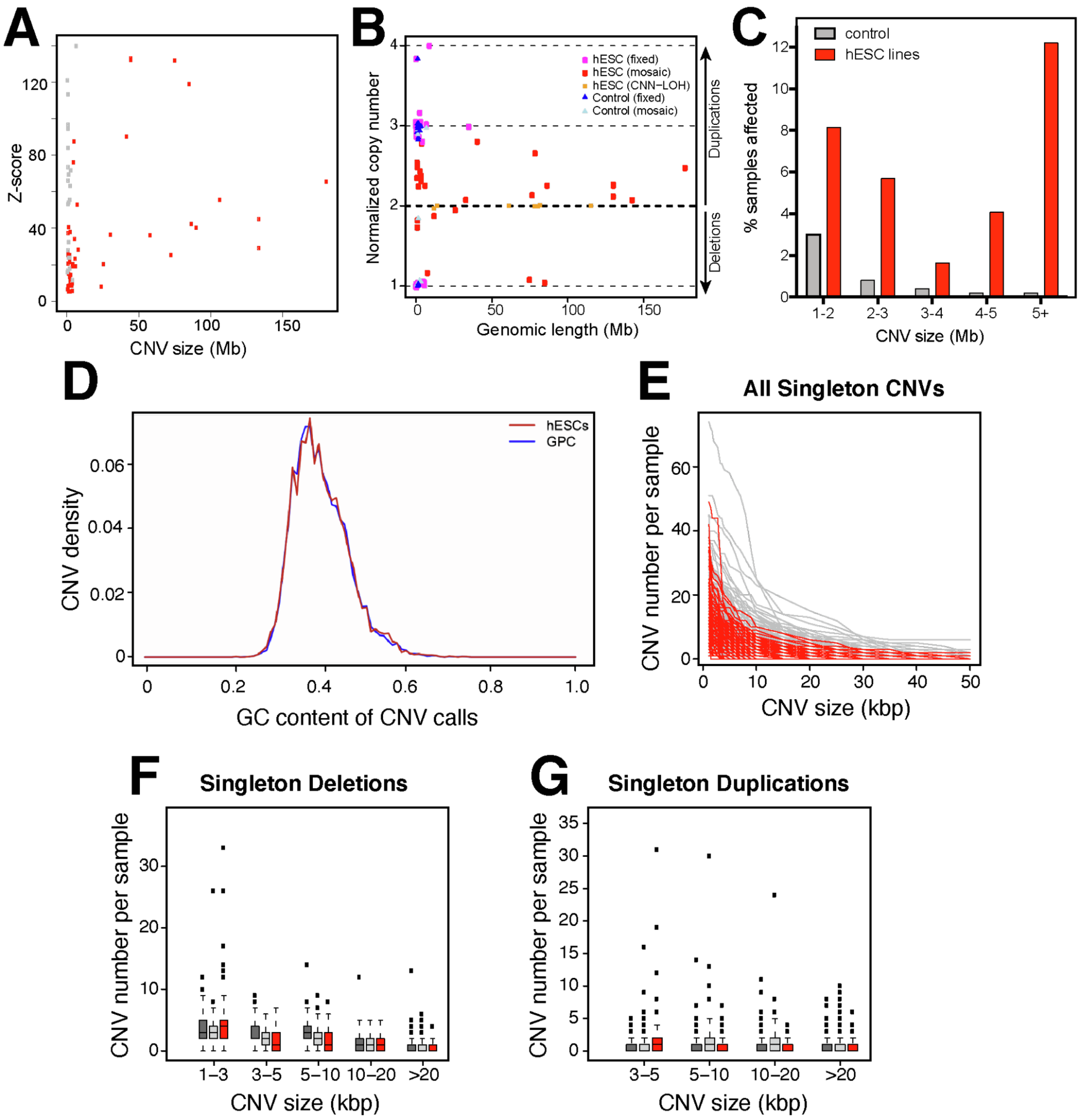

Figure S3: Structural genetic variant calling in hESCs, related to main Figure 4. A) Depth-of-coverage (DOC) analysis enables identification of structural variants over a wide range of sizes based on deviation from expected coverage from other samples considered in parallel (Z-score). Controls are shown in grey and hESCs are shown in red for variants >1 Mbp. **B)** CNVs from hESC or control samples that significantly ($P < 0.001$) deviated from a copy number of 1, 2, 3, or 4 were classified as mosaic. **C)** hESC lines show an excess of CNVs 1 Mbp or larger relative to similarly-sequenced control samples. **D)** Distribution of GC content in CNVs called in hESC WGS libraries prepared by tagmentation (red, hESCs) and CNVs called in control somatic cells from WGS libraries prepared by sonication (blue, GPC). **E)** Analysis of singleton CNVs suggests that hESC samples (red) do not carry an excess burden of potentially culture-acquired small CNVs relative to control populations (grey). **F,G)** Singleton deletions (F) or duplications (G) appearing only once in the dataset are not enriched in hESC lines (red) relative to control African American (dark grey) or Latino (light grey) samples.

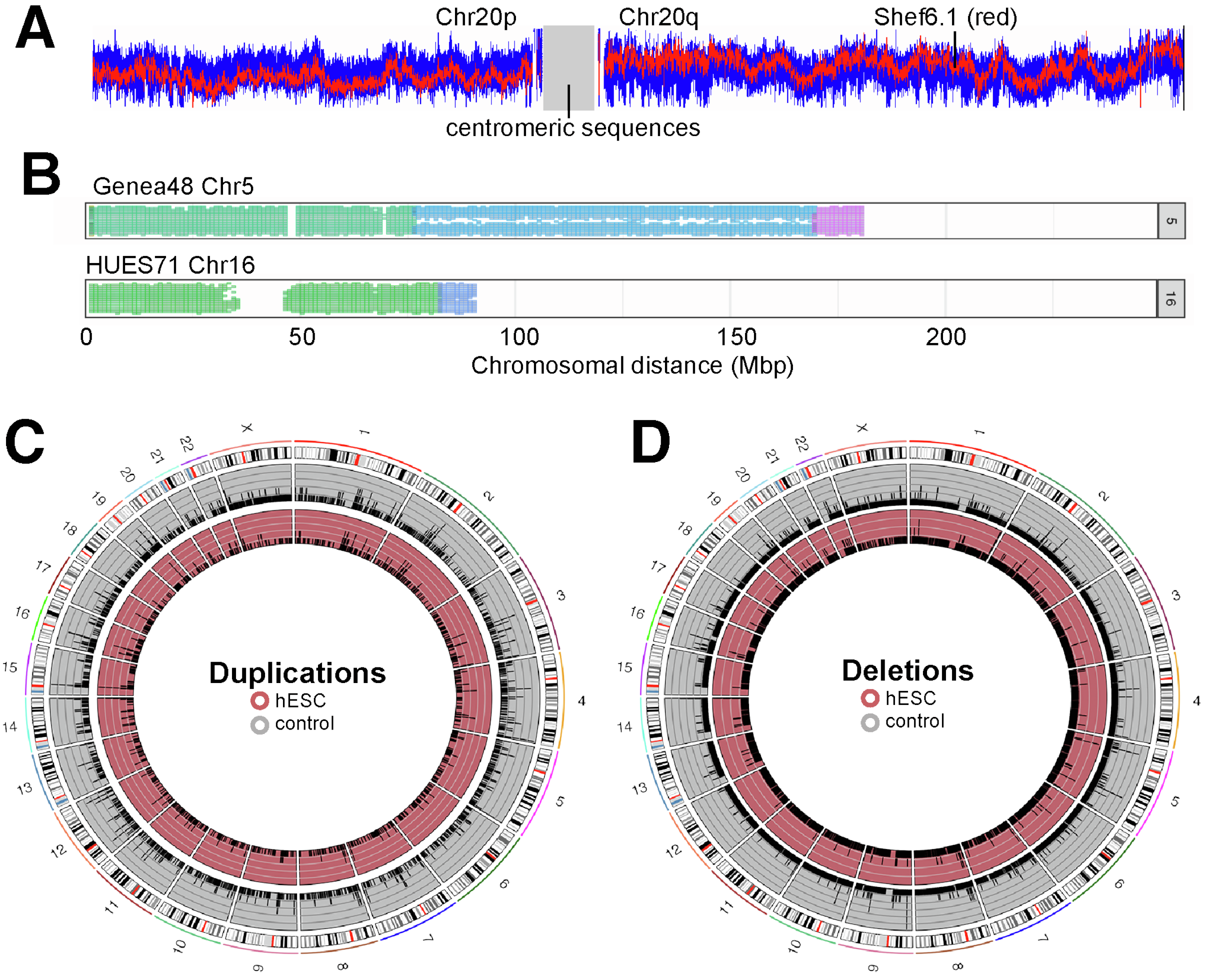

Figure S4: CNV calling and distribution in hESCs, related to main Figure 4. A) Normalized read DOC across chromosome 20 indicates loss of Chr20p and duplication of Chr20q in a subset of cells in hESC line Shef6.1 (red trace) relative to the other 120 cell lines (blue traces). The centromere is indicated in grey. **B)** Two cell lines show evidence for trisomy rescue since they contained genomic regions failed to phase when assuming a diploid model and gave patterns consistent with three distinct haplotypes rather than the expected two haplotypes over sections of chromosome 5 (1,853,207-76,720,244) for Genea48 (top) or chromosome 16 (start-82,687,797) for HUES71 (bottom). The boundaries of these regions likely correspond to recombination and nondisjunction events from meiosis I, which were then resolved in a subset of cells within the blastocyst or in cell culture leading to various levels of mosaicism of these haplotypes in the affected chromosomes (different colours). **C,D)** Small duplications (C) or deletions (D) in hESC lines (red) do not show clear evidence of recurrence relative to control samples (grey).

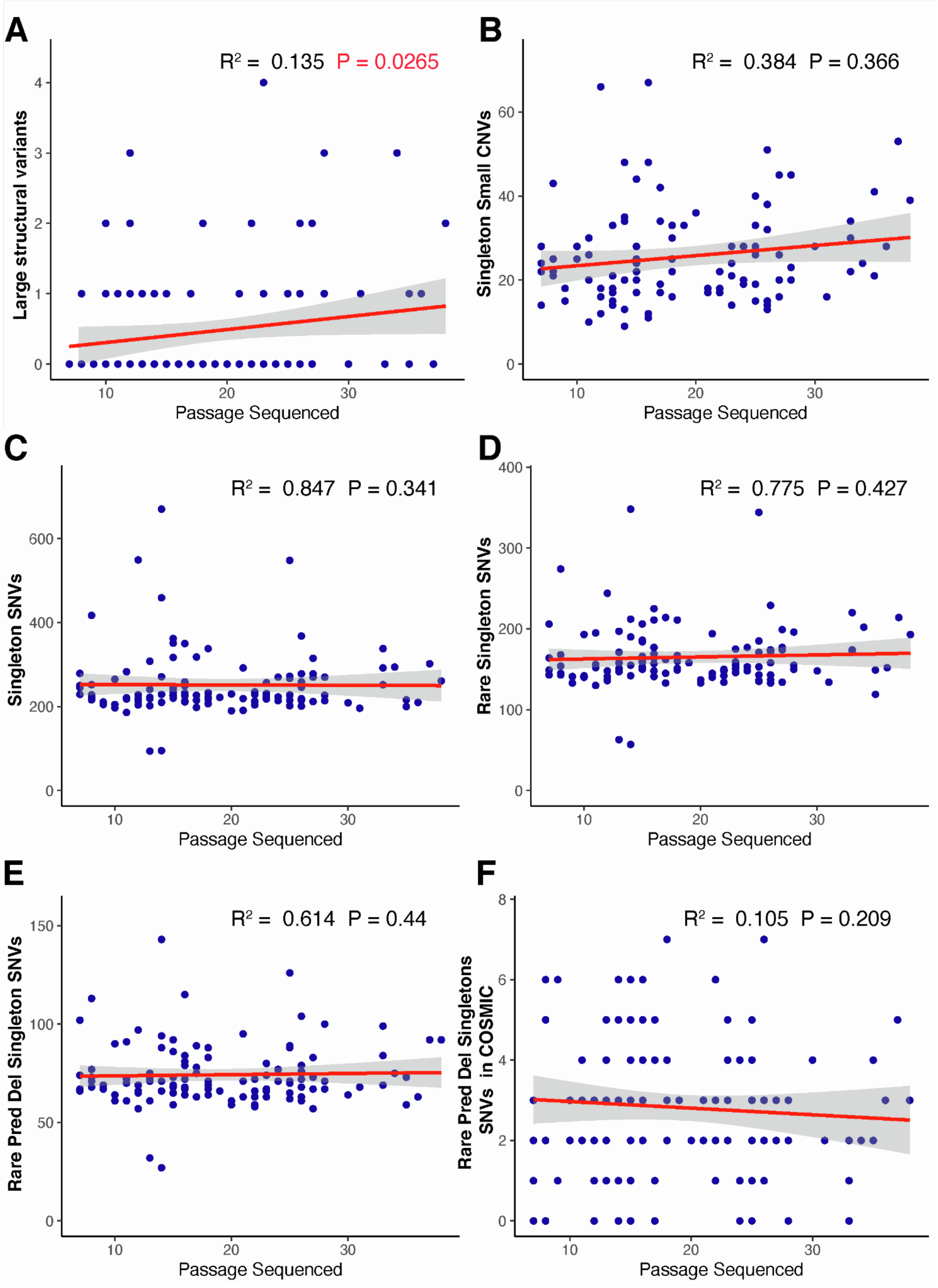

Figure S5: Linear regression across genetic variant types in hESCs, related to main Figures 4-6. The abundance of different classes of genetic variants identified in hESCs was compared to sex chromosome status, clinical-grade or research-grade, passage number at the time of sequencing, and principal components from SNP ancestry analysis. There is a positive correlation between passage number and the abundance of large structural variants per cell line **(A)**, but not for singleton small CNVs (**B**), singleton SNVs (**C**), rare singleton SNVs (**D**), likely deleterious rare singleton SNVs (**E**), or rare SNVs found in the COSMIC database (**F**).
